# Supplementary material for: A HIF–LIMD1 negative feedback mechanism mitigates the pro‐tumorigenic effects of hypoxia
Source: EMBO Mol Med. 2018 Jun 21;10(8):e8304. doi: 10.15252/emmm.201708304 (PMC6079541; doi:10.15252/emmm.201708304)
Supplement: Supplementary file 1 — Appendix [file EMMM-10-e8304-s001.pdf]

Appendix

A HIF-LIMD1 negative feedback mechanism drives the adaptive response to hypoxia and mitigates pro-tumorigenic hypoxic effects

Daniel E. Foxler[1]†, Katherine S. Bridge[1]†, John G. Foster[1], Paul Grevitt[1], Sean Curry[2], Kunal M. Shah[1], Kathryn M. Davidson[1], Ai Nagano[1], Emanuela Gadaleta[1], Hefin I. Rhys[3], Paul T. Kennedy[1], Miguel A. Hermida[1], Ting-Yu Chang[4], Peter E. Shaw[2], Louise E. Reynolds[5], Tristan R. McKay[6], Hsei-Wei Wang[4], Paulo S. Ribeiro[5], Michael J. Plevin[7], Dimitris Lagos[8], Nicholas R. Lemoine[1], Prabhakar Rajan[1], Trevor A. Graham[5], Claude Chelala[1], Kairbaan M. Hodivala-Dilke[5], Ian Spendlove[2] and Tyson V. Sharp[1]*

1. Centre for Molecular Oncology, Barts Cancer Institute, Queen Mary University of London, UK
2. Faculty of Medicine and Life Sciences, University of Nottingham, UK
3. The Francis Crick Institute, London, UK
4. Institute of Microbiology and Immunology, National Yang Ming University, Taiwan
5. Centre for Tumour Biology, Barts Cancer Institute, Queen Mary University of London, UK
6. School of Healthcare Science, Manchester Metropolitan University, UK
7. Department of Biology, University of York, Heslington, York YO10 5DD, UK
8. Centre for Immunology and Infection, Hull York Medical School and Department of Biology, University of York, Heslington, York YO10 5DD, UK

* Corresponding author t.sharp@qmul.ac.uk
† These authors contributed equally to this work

Table of Contents

Appendix Figures S1-S3

Appendix Figure Legends

Appendix Materials and Methods

Appendix Tables S1-S11

Figure S1.

Variable	LIMD1 frequency			VEGF frequency			HIF1a frequency		
	Low	High	P value	Low	High	P value	Low	High	P value
**Age**									
**<70**	55 (28.9%)	37 (19.5%)	0.056	14 (7.0%)	81 (40.7%)	0.051	78 (40.2%)	15 (7.7%)	0.317
**>70**	45 (23.7%)	53 (27.9%)		27 (13.6%)	77 (38.7%)		79 (40.7%)	22 (11.3%)	
**Sex**									
**Male**	48 (24.6%)	45 (23.1%)	0.747	20 (9.9%)	76 (37.4%)	0.831	81 (40.9%)	16 (8.1%)	0.438
**Female**	55 (28.2%)	47 (24.1%)		21 (10.3%)	86 (42.4%)		80 (40.4%)	21 (10.6%)	
**Smoker**									
**Yes**	41 (21.4%)	35 (18.2%)	0.392	21 (10.4%)	63 (31.3%)	0.323	61 (31.1%)	20 (10.2%)	0.215
**Ex**	46 (24.0%)	47 (24.5%)		15 (7.5%)	79 (39.3%)		79 (40.3%)	14 (7.1%)	
**Unknown**	15 (7.8%)	8 (4.2%)		5 (2.5%)	18 (9.0%)		19 (9.7%)	3 (1.5%)	
**Post – Op Histopathology**									
**Adenocarcinoma**	94 (47.2%)	83 (41.7%)	0.846	35 (15.6%)	143 (63.8%)	0.625	143 (65%)	30 (13.6%)	0.178
**Adenosquamous Carcinoma**	8 (4.0%)	9 (4.5%)		6 (2.7%)	18 (8.0%)		17 (7.7%)	7 (3.2%)	
**Other**	3 (1.5%)	2 (1.0%)		3 (1.3%)	19 (8.5%)		21 (9.5%)	2 (0.9%)	
**Differentitation**									
**Well**	8 (4.3%)	14 (7.5%)	0.84	7 (3.6%)	18 (9.2%)	0.492	19 (10.0%)	4 (2.1%)	0.826
**Moderate**	35 (18.7%)	40 (21.4%)		15 (7.7%)	62 (32.8%)		61 (32.1%)	12 (6.3%)	
**Poor**	33 (17.6%)	24 (12.8%)		8 (4.1%)	49 (25.1%)		45 (23.7%)	13 (6.8%)	
**Unknown**	22 (11.8%)	11 (5.9%)		8 (4.1%)	28 (14.4%)		30 (15.8%)	6 (3.2%)	
**Tumour Size**									
**T1a + T1b**	22 (11.3%)	30 (15.5%)	0.22	17 (8.4%)	38 (18.8%)	0.07	39 (19.8%)	13 (6.6%)	0.388
**T2a + T2b**	52 (26.8%)	41 (21.1%)		15 (7.4%)	80 (39.6%)		80 (40.6%)	15 (7.6%)	
**T3 + T4**	28 (14.4%)	21 (10.8%)		9 (4.5%)	43 (21.3%)		41 (20.8%)	9 (4.6%)	
**Node Involvement**									
**0**	56 (29.2%)	56 (29.2%)	0.606	30 (15.0%)	88 (44.0%)	0.114	91 (46.7%)	22 (11.3%)	0.979
**1**	27 (14.1%)	23 (12.0%)		7 (3.5%)	42 (21.0%)		40 (20.5%)	9 (4.6%)	
**2**	18 (9.4%)	12 (6.3%)		4 (2.0%)	29 (14.5%)		27 (13.8%)	6 (3.1%)	
**Metastasis**									
**Yes**	30 (19.6%)	24 (15.7%)	0.403	8 (5.0%)	45 (28.3%)	0.556	48 (31.0%)	5 (3.2%)	0.044
**No**	48 (31.4%)	51 (33.3%)		20 (12.6%)	86 (54.1%)		79 (51.0%)	23 (18.1%)	

Figure S2.

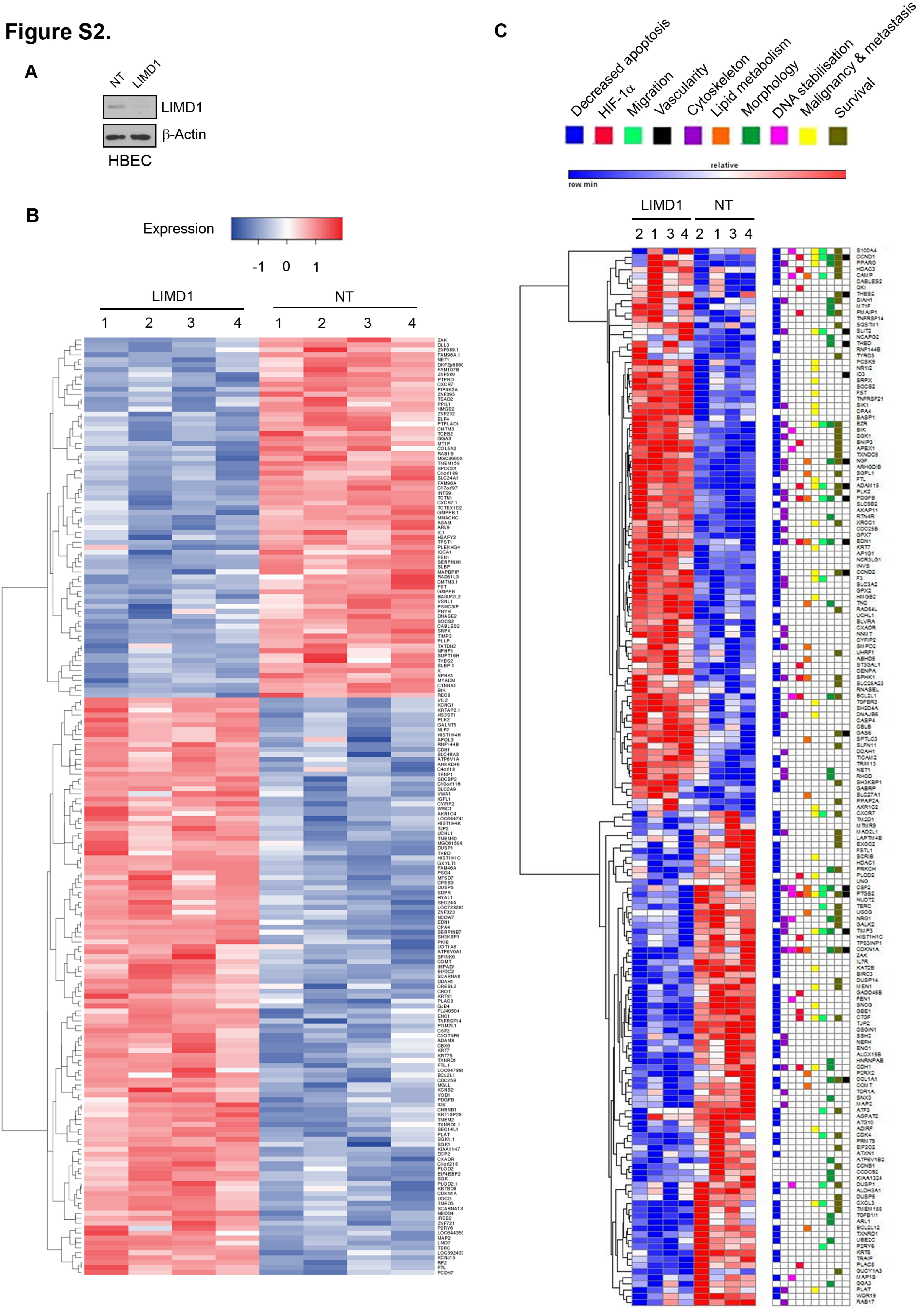
A
NT
LIMD1
LIMD1
β-Actin
HBEC
B
Expression
-1
0
1
LIMD1
NT
1
2
3
4
1
2
3
4
C
Decreased apoptosis
HIF-1α
Migration
Vascularity
Cytoskeleton
Lipid metabolism
Morphology
DNA stabilisation
Malignancy & metastasis
Survival
relative
row min
LIMD1
NT
2
1
3
4
2
1
3
4

Figure S3.

Upstream Regulator	Molecule Type	Predicted Activation State	Activation z-score	p-value of overlap	Target molecules in dataset
HIF1A	transcription regulator	Activated	3.114	1.76E-04	ADAM15,BCL2L1,BNIP3, CCND1,CDH1,CDKN1A,CTGF,EDN1,GADD45B,GBE1, HDAC3,HIST1H1C,PDGFB, PLAC8,PLOD2,PMAIP1, PTGS2,QKI,SPHK1,ST3GAL1

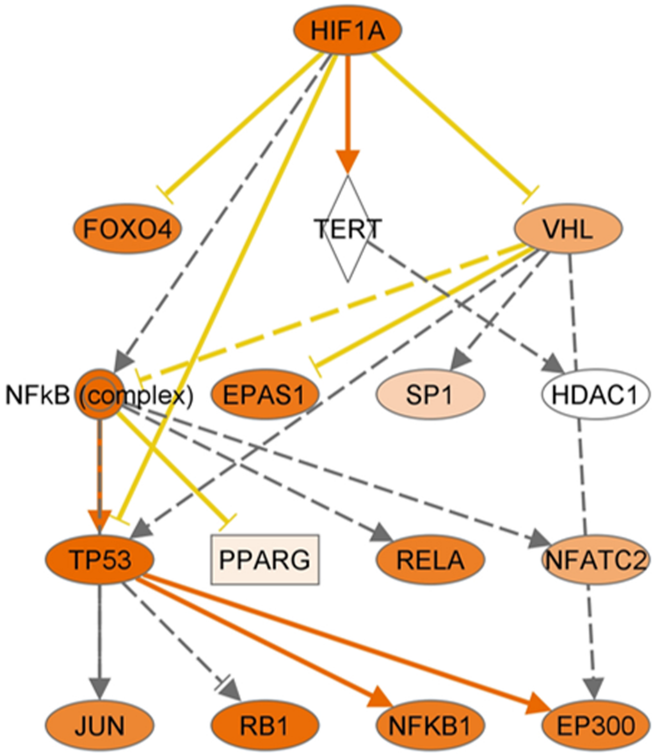

Appendix Figure Legends

Appendix Figure S1

Clinicopathological analysis of LIMD1, VEGF and HIF-1α expressions from analysis of 276 TMA cores from data as described for Fig. 6.

Appendix Figure S2

(A) LIMD1 protein is depleted following siRNA (80nM) treatment of primary human bronchial epithelial (HBEC) cells. Western blot analysis of HBEC cells transfected with 80nm non-targeting (NT) or LIMD1-targeting siRNA for 72 hours.

(B) Heat map analysis of total gene changes following siRNA-mediated depletion of *LIMD1* from HBEC cells. RNA was extracted from NT or LIMD1 siRNA transfected cells as described in (A) and gene expression was quantified by HT12v4.0 Illumina microarray. Heat map displayed is from analysis from 4 technical replicates.

(C) Heat map analysis showing the top 10 altered pathways in primary HBECs following *LIMD1* depletion. Microarray analysed gene expression changes with a q value cut off of <0.15 were interrogated with Ingenuity Pathway Analysis (IPA) software. Gene ontology changes was collected from the Bio Functions read out of IPA results where activation was >+1 or <-1 and categories were collapsed into similar overall functions.

Appendix Figure S3

Identification of HIF-1α regulated ontology gene changes as identified by Ingenuity Pathway Analysis.

Appendix Materials and Methods

Statistical analysis

Figure 1

- **Figure 1A:**

  Data were normalised to each cell line's 0 time point, one-sample t tests were used comparing each mean against the theoretical value of 1.

- **Figure 1C:**

  Data were normalised to each cell line's 0 time point, one-sample t tests were used comparing each mean against the theoretical value of 1. These *p* values had a Holm-Sidak correction applied to them to control the familywise error rate.

- **Figure 1E:**

  Data were normalised to the normoxia groups (0hr), a mixed model ANOVA was performed, allowing for the effect of time to vary between each experiment/replicate. There was a significant effect of Time ($p = 0.005$), Genotype ($p < 0.001$), and an interaction between them ($p = 0.018$).

Figure 2

- **Figure 2C:**

  Data were normalised to the non-targeting (NT) treatment within each oxygen condition (normoxia and hypoxia), other siRNA treatments were compared to NT baseline with one-sample t tests, comparing each mean against the theoretical value of 1. These *p* values had a Holm-Sidak correction applied to them to control the familywise error rate.

Figure 3

- **Figure 3I:**

  Data were normalised to the mean of the 0 hour WT group and analysed using a mixed model ANOVA, where Time and Genotype were entered as fixed factors, and the effect of time was allowed to vary between experimental runs. The model residuals approximated a normal distribution. There was a significant main effect of Genotype ($p = 0.004$), no main effect of Time ($p = 0.113$) but a significant interaction between them ($p = 0.002$) as the difference between the genotypes increases over time. Holm-Sidak post hoc tests of WT vs MT at each level of time demonstrated significance at time 24 hour ($p < 0.001$).

- **Figure 3J:**

  Data were analysed with a 2-way ANOVA where Genotype and Gene were entered as independent variables. There was a significant effect of Genotype, Gene and interaction between them (all $p < 0.001$) as the effect of Genotype was not the same for all genes. The model residuals are approximately normally distributed. Holm-Sidak post-hoc tests comparing WT vs MT at each level of Gene demonstrated significant differences for all comparisons.

- **Figure 3K:**

  Data were analysed using a mixed model ANOVA, where Time and Genotype were entered as fixed factors, and the effect of time was allowed to vary between experimental runs. The model residuals approximated a normal distribution. There was a significant main effect of Genotype ($p = 0.012$), a significant main effect of Time ($p < 0.001$) and a significant interaction between them ($p = 0.044$) as the difference between the genotypes increases over time. Holm-Sidak post hoc tests of WT vs MT at each level of time demonstrated one significant difference, which was at time 48 hours.

Figure 4

- **Figure 4B:**

Densitometry values for HIF-1α protein normalised to β-actin densitometry were compared between 0-30 minutes, and change in densitometry per minute was calculated as rate of decay (ROD) for each genotype (WT/MUT). One-sample t tests were used comparing mean ROD.

- **Figure 4D:**

Data were normalised to the mean of the normoxic WT group. The data were analysed using a mixed model ANOVA, where Time, Genotype and siRNA treatment were entered as fixed factors, and the effect of time was allowed to vary between experimental runs. The model residuals approximated a normal distribution. All three main effects and all four interactions were significant. Holm-Sidak post hoc tests comparing each level of siRNA treatment against every other level, at each combination of Time and Genotype demonstrated only two significant differences: NT vs HIF1a and NT vs HIF2a at Time 48 and MT Genotype only (both $p < 0.001$).

Figure 5

- **Figure 5A:**

Data were analysed using a mixed model ANOVA, where Time and Genotype were entered as fixed factors, and the effect of time was allowed to vary between experimental runs. The model residuals approximated a normal distribution. There was a significant main effect of Genotype, Time, and interaction between them (all $p < 0.001$). Holm-Sidak post hoc tests of WT vs MT at each level of time demonstrated significant differences at each level (all $p < 0.001$).

- **Figure 5B:**

Data were analysed with a 2-way ANOVA where Genotype and Gene were entered as independent variables. There was a significant effect of Genotype, Gene and interaction

between them (all $p < 0.001$) as the effect of Genotype was not the same for all genes. The model residuals are approximately normally distributed. Holm-Sidak post-hoc tests comparing WT vs MT at each level of Gene demonstrated significant differences for VEGF and ALDOC (both $p < 0.001$) only.

- **Figure 5C:**

Data were analysed using a mixed model ANOVA, where Hypoxia and Genotype were entered as fixed factors, and the effect of hypoxia was allowed to vary between experimental runs. The model residuals approximated a normal distribution. There was a significant main effect of genotype ($p = 0.025$) but no main effect of Hypoxia or an interaction. As a result of this, no *post hoc* tests were required, and so the p value of the main effect of genotype is sufficient.

- **Figure 5D:**

One mouse in the WT group with an unusually large tumour volume for its group. Data are analysed with a Mann-Whitney U test which gave a *p* value of 0.005.

- **Figure 5F:**

Data were analysed with a Welch's t test which gave a *p* value of < 0.001 (residuals were approximately normally distributed).

- **Figure 5G-J:**

Distribution of the genotype groups was skewed and the residuals were non-normal, and the groups displayed heterogenous variance, these data were therefore analysed using Welch-corrected t tests applied to log10-transformed data. A linear discriminant analysis was also performed, which is similar to principal components analysis, but finds the linear combination of the log10 expression data which best discriminates the groups. The two groups are completely separated by the single discriminant factor, and the factor loadings

(the correlation of the log10 expression values with the discriminant factor). This shows in a single analysis how the expression values vary across the two groups.

Expanded view Figures

Figure EV1

- **Figure EV1B**

Densitometric analysis of LIMD1 protein normalized to β-actin were normalised to each cell line's 0 time point, one-sample t tests were used comparing each mean against the theoretical value of 1.

- **Figure EV1C-D**

Values for the Time = 0 group of each cell line were equal to 1, therefore the data was analysed with a separate one-sample t test per bar (against the theoretical value of 1) and a Holm-Sidak correction was applied.

- **Figure EV1J**

Data were normalised to vector only (VO) reporter plasmid, and one-sample t tests were used comparing each mean against the theoretical value of 1.

Figure EV2

- **Figure EV2E-I**

Data were normalised to the non-targeting (NT) treatment within each oxygen condition (normoxia and hypoxia), other siRNA treatments were compared to NT baseline with one-sample t tests, comparing each mean against the theoretical value of 1. These *p* values had a Holm-Sidak correction applied to them to control the familywise error rate.

Figure EV3

- **Figure EV3B-E**

Data were analysed using a mixed model ANOVA where Time and Genotype were entered as fixed factors, and the effect of time was allowed to vary between experimental runs. The residuals from both models approximated a normal distribution. *p* values had a Holm-Sidak correction applied to them to control the familywise error rate.

- **Figure EV3F**

Data were analysed using a 2-way ANOVA, entering Time and Genotype as factors. Both main effects and the interation were significant (all $p < 0.001$). The model residuals approximated a normal distribution.

- **Figure EV3G**

Data were analysed with a 2-way ANOVA where Genotype and Time were entered as independent variables. There was a significant effect of Genotype, Time and interaction between them (all $p < 0.001$) as the effect of Genotype was not the same for all levels of Time. The model residuals are approximately normally distributed. Holm-Sidak post-hoc tests compared WT vs MT at each level of Time.

- **Figure EV3H**

Data were analysed with one-sample t tests, comparing the mean of the MT data at each level of Gene against the theoretical value of 1. *p* values then had a Holm-Sidak correction applied to them. The MT data were significantly different from 1 for the ALDOC ($p = 0.042$) and ERO1L ($p = 0.011$) genes, and the HK1 gene was just shy of significance ($p = 0.064$).

- **Figure EV3I**

Data were analysed with a separate one-sample t test per gene (against the theoretical value of 1) and applied a Holm-Sidak correction. The only significant difference was for the HK1 gene (p = 0.031).

- **Figure EV3J**

  Data were analysed with a 2-way ANOVA where Genotype and Gene were entered as independent variables. There was a significant effect of Genotype, Gene and interaction between them (all $p < 0.001$) as the effect of Genotype was not the same for all levels of Gene. The model residuals are approximately normally distributed. Holm-Sidak post-hoc tests comparing WT vs MT at each level of Gene demonstrated significant differences for VEGF ($p < 0.001$), BNIP3 (p = 0.074, almost significant), ALDOC ($p = 0.012$), and HK1 ($p <$ 0.001).

- **Figure EV3K**

  A mixed model ANOVA was applied where Genotype and Gene were entered as independent variables, allowing for a separate intercept for each experimental run. The model residuals were highly heteroscedastic, so a second model was created where the dependent variable was log10 transformed, however the residuals were still heteroscedastic and non-normally distributed. Therefore separate Mann-Whitney U tests were performed, comparing WT and MT at each level of Gene, however there were no significant differences.

- **Figure EV3L**

  Data were analysed using a mixed model ANOVA, where Time and Genotype were entered as fixed factors, and the effect of time was allowed to vary between experimental runs. The model residuals approximated a normal distribution. There were no significant main effects or interactions.

Figure EV4

- **Figure EV4C-O:**

Distribution of the genotype groups was skewed and the residuals were non-normal, and the groups displayed heterogenous variance, these data were therefore analysed using Welch-corrected t tests applied to log10-transformed data. A linear discriminant analysis was also performed, which is similar to principal components analysis, but finds the linear combination of the log10 expression data which best discriminates the groups. The two groups are completely separated by the single discriminant factor, and the factor loadings (the correlation of the log10 expression values with the discriminant factor). This shows in a single analysis how the expression values vary across the two groups.

Table S1: Statistical analysis corresponding to Figure 1

Figure 1A: LIMD1 mRNA

Cell line	1% O2 (h)	N	Mean	SEM
A549	0	3	1	0
	4	3	1.730201	0.050168
	24	3	1.911079	0.169063
	48	3	1.642051	0.276446
HeLa	0	3	1	0
	4	3	2.81952	0.151835
	24	3	3.577857	0.461736
	48	3	3.378797	0.553669
HEK293	0	3	1	0
	4	3	2.963837	0.435978
	24	3	3.753285	0.243858
	48	3	3.487724	0.24913
U2OS	0	3	1	0
	4	3	2.670475	0.072294
	24	3	2.095063	0.113342
	48	3	2.041784	0.045367
SAEC	0	3	1	0
	4	3	1.144392	0.151512
	24	3	1.588118	0.150357
	48	3	1.560516	0.217055
HDF	0	3	1	0
	4	3	0.891052	0.086315
	24	3	1.148354	0.081511
	48	3	1.610363	0.236962

Test Results

Comparison	P value
A549 0h vs. 48h	0.00068
HeLa 0h vs. 48h	5.5E-05
HEK293 0h vs. 48h	1.46E-06
U2OS 0h vs. 48h	0.000134
SAEC 0h vs. 48h	0.042335
HDF 0h vs. 48h	0.039845

Figure 1C: LIMD1 protein densitometry

Cell line	1% O2 (h)	N	Mean	SEM
A549	0	3	1	0
	4	3	1.681685	0.200125
	24	3	2.77541	0.199524
HEK293	0	3	1	0
	4	3	2.049408	0.142132
	24	3	3.020126	0.319687
HeLa	0	3	1	0
	4	3	0.871981	0.12675
	24	3	1.940787	0.293154
U2OS	0	3	1	0
	4	3	0.807274	0.13855
	24	3	1.739663	0.21056

Test Results

Comparison	P value
A549 0h vs. 24h	0.033
HEK293 0h vs. 24h	0.048
HeLa 0h vs. 24h	n.s.
U2OS 0h vs. 24h	n.s.

Figure 1E: Renilla Luciferase/Firefly luciferase activity

Reporter construct	1% O2 (h)	N	Mean	SEM
VO	0		1	0.156446
	4		1.000056	0.084771
	24		1.000058	0.336296
	48		1.000107	0.147003
WT LIMD1 promoter	0		1	0.013527
	4		1.990126	0.230431
	24		3.725871	0.231057
	48		3.481573	0.342414
ΔHRE1	0		1	0.196142
	4		1.814047	0.227388
	24		2.818145	0.580573
	48		3.635332	0.148128
ΔHRE2	0		1	0.18462
	4		1.597805	0.20117
	24		2.524697	0.402358
	48		3.477972	0.679774
ΔHRE3	0		1	0.229593
	4		1.192254	0.06808
	24		0.838765	0.196243
	48		1.492902	0.304928

Test Results

Comparison	P value
VO 0h vs. 48h	n.s.
WT 0h vs. 48h	< 0.001
ΔHRE1 0h vs. 48h	< 0.001
ΔHRE2 0h vs. 48h	< 0.001
ΔHRE3 0h vs. 48h	0.063

Table S2: Statistical analysis corresponding to Figure 2

Figure 2C: LIMD1 mRNA

% O2	siRNA	N	Mean	SEM
20	NT	3	1	
	LIMD1	3	0.5287	0.12503
	PHD2	3	1.61426	0.08549
	HIF-1α	3	0.8364	0.10562
	HIF-2α	3	2.32282	0.03692
1	NT	3	1	
	LIMD1	3	0.44967	0.004637
	PHD2	3	1.789165	0.056872
	HIF-1α	3	0.58448	0.026847
	HIF-2α	3	1.761148	0.204836

Test Results

Comparison	P value
20% O2 NT vs LIMD1	0.058
20% O2 NT vs PHD2	n.s.
20% O2 NT vs HIF-1α	n.s.
20% O2 NT vs HIF-2α	0.098
1% O2 NT vs LIMD1	0.061
1% O2 NT vs PHD2	0.05
1% O2 NT vs HIF-1α	0.004
1% O2 NT vs HIF-2α	0.098

Table S3: Statistical analysis corresponding to Figure 3

Figure 3I: HIF-1 reporter activity (Renilla Luciferase/Firefly luciferase)

1% O2 (h)	LIMD1 promoter genotype	N	Mean	SEM
0	HRE WT	3	1	0.32239
	HRE MUT	3	0.874243	0.202266
4	HRE WT	3	9.825007	0.708228
	HRE MUT	3	10.31575	0.854213
24	HRE WT	3	107.3597	11.31404
	HRE MUT	3	156.8058	10.21354

Test Results

Comparison	P value
0h HRE WT vs MUT	n.s.
4h HRE WT vs MUT	n.s.
24h HRE WT vs MUT	< 0.001

Figure 3J: HIF-1 target gene expression (mRNA)

HIF-1 target gene	LIMD1 promoter genotype	N	Mean	SEM
VEGF	HRE WT	3	0.834597	0.141065
	HRE MUT	3	1.445817	0.015346
BNIP3	HRE WT	3	2.567184	0.121167
	HRE MUT	3	3.486082	0.455913
ALDOC	HRE WT	3	6.233116	0.183827
	HRE MUT	3	10.65871	1.876565
ERO1L	HRE WT	3	1.695276	0.078749
	HRE MUT	3	3.15249	0.292563
HK1	HRE WT	3	1.865569	0.062777
	HRE MUT	3	5.074891	0.737543

Test Results

Comparison	P value
VEGF HRE WT vs MUT	0.015
BNIP3 HRE WT vs MUT	0.001
ALDOC HRE WT vs MUT	< 0.001
ERO1L HRE WT vs MUT	< 0.001
HK1 HRE WT vs MUT	< 0.001

Figure 3K: Secreted VEGF-A

1% O2 (h)	LIMD1 promoter genotype	N	Mean	SEM
0	HRE WT	3	1011.376	4.504147
	HRE MUT	3	1057.897	2.951266
48	HRE WT	3	1226.531	60.04585
	HRE MUT	3	1525.245	51.65455

Test Results

Comparison	P value
0h HRE WT vs MUT	
48h HRE WT vs MUT	

Table S4: Statistical analysis corresponding to Figure 4

Figure 4B: HIF-1α protein rate of decay (ROD)

LIMD1 promoter genotype	N	Mean	SEM
HRE WT	3	0.029291	0.002569
HRE MUT	3	0.017906	0.001047

Test Results	
**Comparison**	**P value**
HRE WT vs MUT	0.014810335

Figure 4D: VEGF mRNA

LIMD1 promoter genotype	1% O2 (h)	siRNA	N	Mean	SEM
HRE WT	0	NT	3	1	0
		HIF-1α	3	1.376	0.430625
		HIF-2α	3	0.992	0.151313
	24	NT	3	3.006	0.404933
		HIF-1α	3	2.373	0.377416
		HIF-2α	3	3.073	0.516907
	48	NT	3	2.61	0.285418
		HIF-1α	3	3.713	0.675008
		HIF-2α	3	2.201	0.363635
HRE MUT	0	NT	3	1.35	0.121175
		HIF-1α	3	1.234	0.208299
		HIF-2α	3	1.267	0.247729
	24	NT	3	4.37	0.850149
		HIF-1α	3	2.677	0.5502
		HIF-2α	3	5.279	1.421119
	48	NT	3	11.57	3.026199
		HIF-1α	3	3.867	0.586527
		HIF-2α	3	3.231	0.395111

Test Results	
**Comparison**	**P value**
48h NT vs HIF-1α	< 0.001
48h NT vs HIF-2α	< 0.001

Figure 4E: Renilla Luciferase/Firefly luciferase activity

1% O2 (h)	LIMD1 genotype	Reporter	N	Mean	SEM
0	LIMD1+/+	VO	3	1	0
		HIF-1α 3'-UTR	3	1.985	0.094285
		HIF-2α 3'-UTR	3	1.098	0.162449
	LIMD1 -/-	VO	3	1	0
		HIF-1α 3'-UTR	3	2.109	0.11414
		HIF-2α 3'-UTR	3	1.228	0.140696
24	LIMD1+/+	VO	3	1	0
		HIF-1α 3'-UTR	3	2.753	0.09806
		HIF-2α 3'-UTR	3	1.59	0.029087
	LIMD1 -/-	VO	3	1	0
		HIF-1α 3'-UTR	3	2.561	0.3195
		HIF-2α 3'-UTR	3	1.72	0.279621

Table S5: Statistical analysis corresponding to Figure 5

Figure 5A: HIF-1 reporter activity (Renilla Luciferase/Firefly luciferase)

1% O2 (h)	LIMD1 promoter genotype	N	Mean	SEM
0	HRE WT	3	1	0.008554
	HRE MUT	3	1.504025	0.004258
4	HRE WT	3	2.151617	0.016207
	HRE MUT	3	3.159257	0.065481
24	HRE WT	3	2.705828	0.013748
	HRE MUT	3	3.632126	0.034875
48	HRE WT	3	16.00696	0.309895
	HRE MUT	3	23.35753	0.690137

Test Results

Comparison	P value
0h HRE WT vs MUT	< 0.001
4h HRE WT vs MUT	< 0.001
24h HRE WT vs MUT	< 0.001
48h HRE WT vs MUT	< 0.001

Figure 5B: HIF-1 target gene expression (mRNA)

HIF-1 target gene	LIMD1 promoter genotype	N	Mean	SEM
VEGF	HRE WT	3	3.225238	0.358721
	HRE MUT	3	7.184048	0.836913
BNIP3	HRE WT	3	6.266684	0.262935
	HRE MUT	3	7.189146	0.902798
PHD2	HRE WT	3	1.884834	0.057579
	HRE MUT	3	3.242134	0.066034
ALDOC	HRE WT	3	18.93575	4.245962
	HRE MUT	3	29.0115	1.140679
ERO1L	HRE WT	3	1.963057	0.027365
	HRE MUT	3	3.04568	0.014153
HK1	HRE WT	3	1.200349	0.107589
	HRE MUT	3	2.732961	0.29073

Test Results

Comparison	P value
VEGF HRE WT vs MUT	< 0.001
BNIP3 HRE WT vs MUT	n.s.
PHD2 HRE WT vs MUT	n.s.
ALDOC HRE WT vs MUT	< 0.001
ERO1L HRE WT vs MUT	n.s.
HK1 HRE WT vs MUT	n.s.

Figure 5C: Secreted VEGF-A

1% O2 (h)	LIMD1 promoter genotype	N	Mean	SEM
0	HRE WT	3	287.012	16.08936
	HRE MUT	3	309.225	8.524913
48	HRE WT	3	439.351	4.404423
	HRE MUT	3	498.88	38.11353

Test Results

Comparison	P value
0h HRE WT vs MUT	n.s.
48h HRE WT vs MUT	0.025

Figure 5D: Tumour volume

LIMD1 promoter genotype	N	Mean	Plot centile
HRE WT	23	454.8569	5-95
HRE MUT	24	598.0831	5-95

Test Results

Comparison	P value
HRE WT vs MUT	0.005

Figure 5F: Blood Vessel Density

LIMD1 promoter genotype	N	Mean	Plot centile
HRE WT	23	10.24788	5-95
HRE MUT	24	13.63644	5-95

Test Results

Comparison	P value
HRE WT vs MUT	< 0.001

Figure 5G: Endomucin mRNA expression

LIMD1 promoter genotype	N	Mean
HRE WT	15	6.302935
HRE MUT	14	9.988339

Test Results

Comparison	P value
HRE WT vs MUT	0.0018

Figure 5H: VEGF-A mRNA expression

LIMD1 promoter genotype	N	Mean
HRE WT	15	0.050267
HRE MUT	14	0.079286

Test Results

Comparison	P value
HRE WT vs MUT	0.038

Figure 5I: HK1 mRNA expression

LIMD1 promoter genotype	N	Mean
HRE WT	15	0.14703
HRE MUT	14	0.394601

Test Results

Comparison	P value
HRE WT vs MUT	0.0033

Figure 5J: PDK1 mRNA expression

LIMD1 promoter genotype	N	Mean
HRE WT	15	2.079316
HRE MUT	14	3.901869

Test Results

Comparison	P value
HRE WT vs MUT	0.011

Table S6: Statistical analysis corresponding to Figure EV1

Figure EV1B: LIMD1 protein densitometry

Cell line	1% O2 (h)	N	Mean	SEM
SAEC	0		1	0
	4		0.814549474	0.203549
	8		1.444032805	0.340638
	16		1.647395773	0.150907
	24		2.224609207	0.208663
	48		2.919424007	0.29907
HDF	0		1	0
	4		1.165514296	0.236183
	8		1.317969226	0.296096
	16		1.687970809	0.271558
	24		1.846418628	0.293968
	48		2.238126911	0.301914

Test Results

Comparison	P value
SAEC 0h vs. 48h	0.022657
HDF 0h vs. 48h	0.026235

Figure EV1C: PHD2 mRNA

Cell line	1% O2 (h)	N	Mean	SEM
A549	0	3	1	0.087979
	4	3	1.730201	0.050168
	24	3	1.911079	0.169063
	48	3	1.642051	0.276446
HeLa	0	3	1	0.045582
	4	3	2.81952	0.151835
	24	3	3.577857	0.461736
	48	3	3.378797	0.553669
HEK293	0	3	1	0.00631
	4	3	2.963837	0.435978
	24	3	3.753285	0.243858
	48	3	3.487724	0.24913
U2OS	0	3	1	0.01183
	4	3	2.670475	0.072294
	24	3	2.095063	0.113342
	48	3	2.041784	0.045367

Test Results

Comparison	P value
A549 0h vs. 48h	0.042
HeLa 0h vs. 48h	0.027
HEK293 0h vs. 48h	0.005
U2OS 0h vs. 48h	0.032

Figure EV1D: PHD1 mRNA

Cell line	1% O2 (h)	N	Mean	SEM
A549	0	3	1	0.251317
	4	3	1.137757	0.010567
	24	3	1.168199	0.083954
	48	3	1.219928	0.007718
HeLa	0	3	1	0.146334
	4	3	1.014056	0.050019
	24	3	0.694706	0.02901
	48	3	1.119545	0.035926
HEK293	0	3	1	0.066921
	4	3	1.168925	0.403475
	24	3	1.043164	0.09847
	48	3	0.79594	0.154276
U2OS	0	3	1	0.215434
	4	3	1.034054	0.195074
	24	3	0.928844	0.09139
	48	3	0.867316	0.050044

Table S6 continued: Statistical analysis corresponding to Figure EV1

Figure EV1E:	PHD2 protein densitometry			
	Cell line	1% O2 (h)	N	Value
	A549	0	1	1
		4	1	2.288695
		24	1	3.030097
	HeLa	0	1	1
		4	1	2.487166
		24	1	2.554849
	HEK293	0	1	1
		4	1	2.496161
		24	1	3.693751
	U2OS	0	1	1
		4	1	2.241257
		24	1	4.015522

Figure EV1F:	HIF-1α protein densitometry			
	Cell line	1% O2 (h)	N	Value
	A549	0	1	1
		4	1	2.361423
		24	1	0.908581
	HeLa	0	1	1
		4	1	5.285539
		24	1	1.666913
	HEK293	0	1	1
		4	1	5.113687
		24	1	8.229166
	U2OS	0	1	1
		4	1	2.479092
		24	1	0.807874

Figure EV1G:	HIF-2α protein densitometry			
	Cell line	1% O2 (h)	N	Value
	A549	0	1	1
		4	1	6.111773
		24	1	1.361769
	HeLa	0	1	1
		4	1	3.150834
		24	1	2.536862
	HEK293	0	1	1
		4	1	8.904363
		24	1	8.133206
	U2OS	0	1	1
		4	1	0.782921
		24	1	0.524142

Figure EV1J:	Renilla Luciferase/Firefly luciferase activity			
	Reporter construct	N	Mean	SEM
	VO	3	1.000058	0.336296
	WT LIMD1 promoter	3	3.725871	0.231057
	Δ1	3	2.074619	0.064489
	Δ2	3	2.699325	0.11087
	Δ3	3	1.254855	0.200267
	Δ4	3	2.651687	0.122075
	Δ5	3	2.883119	0.196423
	Δ6	3	2.537705	0.306687
	Δ7	3	2.85969	0.451653
	Δ8	3	2.350204	0.159177
	Δ9	3	2.465529	0.110371
	Δ10	3	2.867579	0.146105

Table S7: Statistical analysis corresponding to Figure EV2

Figure EV2A: Renilla Luciferase/Firefly luciferase activity

1% O2 (h)	shRNA	N	Mean	SEM
0	shSCR	3	1	0
	shHIF-1α	3	1	0
	shHIF-2α	3	1	0
4	shSCR	3	16.455	2.574285
	shHIF-1α	3	4.50364	1.532525
	shHIF-2α	3	24.11767	3.173892
8	shSCR	3	37.49693	3.034482
	shHIF-1α	3	7.724148	1.236423
	shHIF-2α	3	47.52997	4.51451
24	shSCR	3	72.61085	6.055389
	shHIF-1α	3	16.67209	2.191585
	shHIF-2α	3	86.37336	8.205971

Figure EV2E: HeLa LIMD1 mRNA

% O2	siRNA	N	Mean	SEM
20	NT	3	1	0
	LIMD1	3	0.430891	0.081721
	PHD2	3	1.192083	0.107235
	HIF-1α	3	1.024563	0.054263
	HIF-2α	3	1.265639	0.107171
1	NT	3	1	
	LIMD1	3	0.455398	0.068415
	PHD2	3	1.644004	0.547063
	HIF-1α	3	0.329133	0.017432
	HIF-2α	3	1.399395	0.022244

Test Results

Comparison	P value
20% O2 NT vs LIMD1	0.0031
20% O2 NT vs PHD2	n.s.
20% O2 NT vs HIF-1α	n.s.
20% O2 NT vs HIF-2α	n.s.
1% O2 NT vs LIMD1	0.0106
1% O2 NT vs PHD2	n.s.
1% O2 NT vs HIF-1α	0.0035
1% O2 NT vs HIF-2α	n.s.

Figure EV2F: U2OS HIF-1α mRNA

% O2	siRNA	N	Mean	SEM
20	NT	3	1	0
	LIMD1	3	0.832197	0.066773
	PHD2	3	0.722169	0.006445
	HIF-1α	3	0.081694	0.001458
	HIF-2α	3	0.894843	0.059853
1	NT	3	1	0
	LIMD1	3	1.045075	0.004664
	PHD2	3	1.021964	0.013681
	HIF-1α	3	0.086137	0.001922
	HIF-2α	3	0.917288	0.01228

Test Results

Comparison	P value
20% O2 NT vs LIMD1	n.s.
20% O2 NT vs PHD2	n.s.
20% O2 NT vs HIF-1α	0.001
20% O2 NT vs HIF-2α	n.s.
1% O2 NT vs LIMD1	n.s.
1% O2 NT vs PHD2	n.s.
1% O2 NT vs HIF-1α	0.001
1% O2 NT vs HIF-2α	n.s.

Table S7 continued: Statistical analysis corresponding to Figure EV2

Figure EV2G: U2OS HIF-2α mRNA

% O2	siRNA	N	Mean	SEM
20	NT	3	1	0
	LIMD1	3	0.820589	0.031927
	PHD2	3	0.556566	0.045975
	HIF-1α	3	0.660805	0.028922
	HIF-2α	3	0.148344	0
1	NT	3	1	
	LIMD1	3	0.594552	0.011568
	PHD2	3	0.447945	0.032658
	HIF-1α	3	0.557362	0.010845
	HIF-2α	3	0.067357	0.008497

Test Results

Comparison	P value
20% O2 NT vs LIMD1	0.097
20% O2 NT vs PHD2	0.022
20% O2 NT vs HIF-1α	0.055
20% O2 NT vs HIF-2α	0.008
1% O2 NT vs LIMD1	0.055
1% O2 NT vs PHD2	0.015
1% O2 NT vs HIF-1α	0.022
1% O2 NT vs HIF-2α	0.003

Figure EV2H: HeLa HIF-1α mRNA

% O2	siRNA	N	Mean	SEM
20	NT	3	1	0
	LIMD1	3	0.977814	0.190766
	PHD2	3	0.733831	0.155992
	HIF-1α	3	0.142944	0.005102
	HIF-2α	3	0.937373	0.033456
1	NT	3	1	0
	LIMD1	3	1.940898	0.361651
	PHD2	3	1.532716	0.43188
	HIF-1α	3	0.196936	0.010541
	HIF-2α	3	1.140575	0.00509

Test Results

Comparison	P value
20% O2 NT vs LIMD1	n.s.
20% O2 NT vs PHD2	0.078
20% O2 NT vs HIF-1α	0.001
20% O2 NT vs HIF-2α	n.s.
1% O2 NT vs LIMD1	0.074
1% O2 NT vs PHD2	0.055
1% O2 NT vs HIF-1α	0.002
1% O2 NT vs HIF-2α	0.078

Figure EV2I: HeLa HIF-2α mRNA

% O2	siRNA	N	Mean	SEM
20	NT	3	1	0
	LIMD1	3	0.575096	0.108459
	PHD2	3	0.320513	0.023367
	HIF-1α	3	0.402389	0.0137
	HIF-2α	3	0.122585	0.008937
1	NT	3	1	0
	LIMD1	3	1.676209	0.130336
	PHD2	3	1.15528	0.112224
	HIF-1α	3	0.586705	0.048465
	HIF-2α	3	0.163349	0.005561

Test Results

Comparison	P value
20% O2 NT vs LIMD1	n.s.
20% O2 NT vs PHD2	0.005
20% O2 NT vs HIF-1α	0.044
20% O2 NT vs HIF-2α	0.003
1% O2 NT vs LIMD1	0.039
1% O2 NT vs PHD2	0.046
1% O2 NT vs HIF-1α	0.036
1% O2 NT vs HIF-2α	0.005

Table S8: Statistical analysis corresponding to Figure EV3

Figure EV3B: U2OS LIMD1 protein densitometry

1% O2 (h)	LIMD1 promoter genotype	N	Mean	SEM
0	HRE WT	3	1	0
	HRE MUT	3	0.968741	0.090075
4	HRE WT	3	1.457119	0.405613
	HRE MUT	3	1.054535	0.239482
24	HRE WT	3	2.04019	0.366225
	HRE MUT	3	0.939389	0.329581
48	HRE WT	3	2.592545	0.338275
	HRE MUT	3	0.646413	0.241377

Test Results	
**Comparison**	**P value**
0h HRE WT vs MUT	n.s.
4h HRE WT vs MUT	n.s.
24h HRE WT vs MUT	n.s.
48h HRE WT vs MUT	< 0.001

Figure EV3C: U2OS Flag protein densitometry

1% O2 (h)	LIMD1 promoter genotype	N	Mean	SEM
0	HRE WT	3	1	0
	HRE MUT	3	0.882484	0.133817
4	HRE WT	3	1.511969	0.272387
	HRE MUT	3	1.324088	0.22777
24	HRE WT	3	1.87307	0.117384
	HRE MUT	3	1.406213	0.163232
48	HRE WT	3	2.268933	0.243158
	HRE MUT	3	1.244311	0.086561

Test Results	
**Comparison**	**P value**
0h HRE WT vs MUT	n.s.
4h HRE WT vs MUT	n.s.
24h HRE WT vs MUT	n.s.
48h HRE WT vs MUT	< 0.001

Figure EV3D: HeLa LIMD1 protein densitometry

1% O2 (h)	LIMD1 promoter genotype	N	Mean	SEM
0	HRE WT	3	1	0
	HRE MUT	3	1	0
4	HRE WT	3	1.892217	0.229249
	HRE MUT	3	1.531759	0.455872
8	HRE WT	3	2.101769	0.558619
	HRE MUT	3	1.807513	0.604396
16	HRE WT	3	1.990277	0.612288
	HRE MUT	3	1.962912	0.599207
24	HRE WT	3	3.203586	0.294965
	HRE MUT	3	2.038284	0.318379
48	HRE WT	3	2.469491	0.614722
	HRE MUT	3	1.317699	0.450292

Test Results	
**Comparison**	**P value**
0h HRE WT vs MUT	n.s.
4h HRE WT vs MUT	0.433
8h HRE WT vs MUT	0.113
16h HRE WT vs MUT	0.914
24h HRE WT vs MUT	0.014
48h HRE WT vs MUT	0.020

Figure EV3E: SAEC LIMD1 protein densitometry

1% O2 (h)	LIMD1 promoter genotype	N	Mean	SEM
0	HRE WT	4	1	0
	HRE MUT	4	0.979634	0.200951
4	HRE WT	4	1.628498	0.507424
	HRE MUT	4	1.577723	0.210042
24	HRE WT	4	1.448758	0.337276
	HRE MUT	4	1.184112	0.280129
16	HRE WT	4	2.337482	0.613342
	HRE MUT	4	1.244539	0.251227
24	HRE WT	4	4.309099	0.780332
	HRE MUT	4	2.199186	0.265905
48	HRE WT	4	3.851951	0.969686
	HRE MUT	4	1.861908	0.232557

Test Results	
**Comparison**	**P value**
0h HRE WT vs MUT	0.461
4h HRE WT vs MUT	0.465
8h HRE WT vs MUT	0.419
16h HRE WT vs MUT	0.075
24h HRE WT vs MUT	0.021
48h HRE WT vs MUT	0.031

Table S8 continued: Statistical analysis corresponding to Figure EV3

Figure EV3F: HeLa HIF-1 mRNA

1% O2 (h)	LIMD1 promoter genotype	N	Mean	SEM
0	HRE WT	3	5.614191	0.77417
	HRE MUT	3	4.262376	0.644226
4	HRE WT	3	4.891543	0.847023
	HRE MUT	3	6.353057	0.819861
24	HRE WT	3	7.385615	0.164774
	HRE MUT	3	9.081321	0.243118

Test Results

Comparison	P value
0h HRE WT vs MUT	n.s.
4h HRE WT vs MUT	n.s.
24h HRE WT vs MUT	<0.001

Figure EV3G: HeLa HIF-1 reporter activity

1% O2 (h)	LIMD1 promoter genotype	N	Mean	SEM
0	HRE WT	3	1	0.119612
	HRE MUT	3	0.950657	0.126442
4	HRE WT	3	6.220234	0.791379
	HRE MUT	3	6.809766	0.423903
24	HRE WT	3	89.25114	4.627653
	HRE MUT	3	135.7146	11.51675

Test Results

Comparison	P value
0h HRE WT vs MUT	n.s.
4h HRE WT vs MUT	n.s.
24h HRE WT vs MUT	<0.001

Figure EV3H: HIF-1 target gene expression (mRNA) 20% O2 U2OS

HIF-1 target gene	LIMD1 promoter genotype	N	Mean	SEM
VEGF	HRE WT	3	1	0.184695
	HRE MUT	3	1.040703	0.132202
BNIP3	HRE WT	3	1	0.041958
	HRE MUT	3	1.062439	0.089076
ALDOC	HRE WT	3	1	0.018656
	HRE MUT	3	1.188325	0.040885
ERO1L	HRE WT	3	1	0.125137
	HRE MUT	3	1.20204	0.105991
HK1	HRE WT	3	1	0.050464
	HRE MUT	3	1.292022	0.057961

Test Results

Comparison	P value
VEGF HRE WT vs MUT	n.s.
BNIP3 HRE WT vs MUT	n.s.
ALDOC HRE WT vs MUT	0.042
ERO1L HRE WT vs MUT	0.011
HK1 HRE WT vs MUT	n.s.

Table S8 continued: Statistical analysis corresponding to Figure EV3

Figure EV3I: HIF-1 target gene expression (mRNA) 20% O2 HeLa

HIF-1 target gene	LIMD1 promoter genotype	N	Mean	SEM
VEGF	HRE WT	3	1	0.058347
	HRE MUT	3	0.977678	0.062224
BNIP3	HRE WT	3	1	0.146368
	HRE MUT	3	1.201496	0.025211
ALDOC	HRE WT	3	1	0.024578
	HRE MUT	3	1.052268	0.005173
ERO1L	HRE WT	3	1	0.04181
	HRE MUT	3	1.124445	0.073101
HK1	HRE WT	3	1	0.005609
	HRE MUT	3	1.458345	0.024541

Test Results

Comparison	P value
VEGF HRE WT vs MUT	n.s.
BNIP3 HRE WT vs MUT	n.s.
ALDOC HRE WT vs MUT	n.s.
ERO1L HRE WT vs MUT	n.s.
HK1 HRE WT vs MUT	0.031

Figure EV3J: HIF-1 target gene expression (mRNA) 1% O2 HeLa

HIF-1 target gene	LIMD1 promoter genotype	N	Mean	SEM
VEGF	HRE WT	3	1.269612	0.094248
	HRE MUT	3	1.988737	0.200202
BNIP3	HRE WT	3	1.6748	0.096597
	HRE MUT	3	1.930247	0.101219
ALDOC	HRE WT	3	1.580928	0.038856
	HRE MUT	3	1.955033	0.211031
ERO1L	HRE WT	3	1.668492	0.023259
	HRE MUT	3	1.810212	0.008412
HK1	HRE WT	3	2.279292	0.556926
	HRE MUT	3	3.558991	0.358745

Test Results

Comparison	P value
VEGF HRE WT vs MUT	<0.001
BNIP3 HRE WT vs MUT	n.s.
ALDOC HRE WT vs MUT	0.012
ERO1L HRE WT vs MUT	n.s.
HK1 HRE WT vs MUT	<0.001

Figure EV3K: HIF-1 target gene expression (mRNA) 1% O2 SAEC

HIF-1 target gene	LIMD1 promoter genotype	N	Mean	SEM
VEGF	HRE WT	3	1.000	0.010
	HRE MUT	3	1.359	0.100
BNIP3	HRE WT	3	1.000	0.010
	HRE MUT	3	1.240	0.050
ALDOC	HRE WT	3	1.000	0.010
	HRE MUT	3	1.230	0.050
ERO1L	HRE WT	3	1.000	0.010
	HRE MUT	3	1.090	0.050
SLC2A1	HRE WT	3	1.000	0.030
	HRE MUT	3	1.230	0.050
GAPDH	HRE WT	3	1.000	0.030
	HRE MUT	3	1.220	0.080
PGM2	HRE WT	3	1.000	0.010
	HRE MUT	3	1.120	0.050
ALDOA	HRE WT	3	1.000	0.020
	HRE MUT	3	1.230	0.030
LDHA	HRE WT	3	1.000	0.010
	HRE MUT	3	1.170	0.030
PHD2	HRE WT	3	1.000	0.020
	HRE MUT	3	1.370	0.080

Test Results

Comparison	P value
VEGF HRE WT vs MUT	n.s.
BNIP3 HRE WT vs MUT	n.s.
ALDOC HRE WT vs MUT	0.057
ERO1L HRE WT vs MUT	n.s.
SLC2A1 HRE WT vs MUT	0.052
GAPDH HRE WT vs MUT	0.086
PGM2 HRE WT vs MUT	0.081
ALDOA HRE WT vs MUT	n.s.
LDHA HRE WT vs MUT	0.045
PHD2 HRE WT vs MUT	0.052

Figure EV3L: HeLa Secreted VEGF-A

1% O2 (h)	LIMD1 promoter genotype	N	Mean	SEM
0	HRE WT	3	1171.171	22.93967
	HRE MUT	3	1144.058	44.47499
48	HRE WT	3	1322.891	19.60103
	HRE MUT	3	1515.217	25.66618

Test Results

Comparison	P value
0h HRE WT vs MUT	n.s.
48h HRE WT vs MUT	n.s.

Table S9: Statistical analysis corresponding to Figure EV4

Figure EV4A: **Number of cells x 10^4**

% O2	LIMD1 promoter genotype	Days	N	Mean	SEM
20	HRE WT	0	3	0.05	0
		3	3	0.37135	0.017135
		5	3	1.8225	0.128849
		7	3	6.102	0.355286
	HRE MUT	0	3	0.05	0
		3	3	0.26865	0.013098
		5	3	1.575	0.05592
		7	3	5.21	0.141778
1	HRE WT	0	3	0.05	0
		3	3	0.2385	0.024943
		5	3	0.848333	0.030322
		7	3	1.443	0.101838
	HRE MUT	0	3	0.05	0
		3	3	0.1988	0.021545
		5	3	0.795	0.077015
		7	3	1.509	0.056338

Test Results

Comparison	P value
20% 0 days HRE WT vs MUT	n.s.
20% 3 days HRE WT vs MUT	n.s.
20% 5 days HRE WT vs MUT	n.s.
20% 7 days HRE WT vs MUT	n.s.
1% 0 days HRE WT vs MUT	n.s.
1% 3 days HRE WT vs MUT	n.s.
1% 5 days HRE WT vs MUT	n.s.
1% 7 days HRE WT vs MUT	n.s.

Figure EV4B: **Number of colonies**

% O2	LIMD1 promoter genotype	N	Mean	SEM
20	HRE WT	3	65.3287	4.309052
	HRE MUT	3	71.4838	3.2775
1	HRE WT	3	68.37269	5.255744
	HRE MUT	3	76.63195	2.030395

Test Results

Comparison	P value
20% HRE WT vs MUT	n.s.
1% HRE WT vs MUT	n.s.

Figure EV4C: **VEGF-B mRNA expression**

LIMD1 promoter genotype	N	Mean
HRE WT	15	0.937977
HRE MUT	14	2.678546

Test Results

Comparison	P value
HRE WT vs MUT	0.014

Figure EV4D: **VEGFC mRNA expression**

LIMD1 promoter genotype	N	Mean
HRE WT	15	0.738175
HRE MUT	14	1.46448

Test Results

Comparison	P value
HRE WT vs MUT	0.011

Figure EV4E: **BNIP3 mRNA expression**

LIMD1 promoter genotype	N	Mean
HRE WT	15	0.074807
HRE MUT	14	0.130271

Test Results

Comparison	P value
HRE WT vs MUT	0.015

Table S9 continued: Statistical analysis corresponding to Figure EV4

Figure EV4F: **PDGFB mRNA expression**

LIMD1 promoter genotype	N	Mean
HRE WT	15	1.507255
HRE MUT	14	2.175619

Test Results	
**Comparison**	**P value**
HRE WT vs MUT	0.032

Figure EV4G: **HIF-1α mRNA expression**

LIMD1 promoter genotype	N	Mean
HRE WT	15	3.017813
HRE MUT	14	5.352436

Test Results	
**Comparison**	**P value**
HRE WT vs MUT	0.073

Figure EV4H: **HIF-2α mRNA expression**

LIMD1 promoter genotype	N	Mean
HRE WT	15	0.024033
HRE MUT	14	0.047921

Test Results	
**Comparison**	**P value**
HRE WT vs MUT	0.0042

Figure EV4I: **ENO1 mRNA expression**

LIMD1 promoter genotype	N	Mean
HRE WT	15	5.247372
HRE MUT	14	10.23965

Test Results	
**Comparison**	**P value**
HRE WT vs MUT	0.0013

Figure EV4J: **EDN1**

LIMD1 promoter genotype	N	Mean
HRE WT	15	3.0722
HRE MUT	14	5.531214

Test Results	
**Comparison**	**P value**
HRE WT vs MUT	0.0047

Figure EV4K: **ALDOC mRNA expression**

LIMD1 promoter genotype	N	Mean
HRE WT	15	0.088881
HRE MUT	14	0.128143

Test Results	
**Comparison**	**P value**
HRE WT vs MUT	0.07

Figure EV4L: **SOD2 mRNA expression**

LIMD1 promoter genotype	N	Mean
HRE WT	15	1.1048
HRE MUT	14	1.793857

Test Results	
**Comparison**	**P value**
HRE WT vs MUT	0.061

Figure EV4M: **KITLG mRNA expression**

LIMD1 promoter genotype	N	Mean
HRE WT	15	3.615933
HRE MUT	14	6.701643

Test Results	
**Comparison**	**P value**
HRE WT vs MUT	0.00066

Figure EV4N: **SLC2A1 mRNA expression**

LIMD1 promoter genotype	N	Mean
HRE WT	15	11.15864
HRE MUT	14	21.58199

Test Results	
**Comparison**	**P value**
HRE WT vs MUT	0.0012

Figure EV4O: **SLC2A14**

LIMD1 promoter genotype	N	Mean
HRE WT	15	5.468563
HRE MUT	14	7.396154

Test Results	
**Comparison**	**P value**
HRE WT vs MUT	0.036

Table S10: Primers

LIMD1 promoter HRE mutagenesis primers

		Sequence
**ΔHRE1**	**Forward**	CCTCTACGAATAACGAGCCTACTAGGGTGTATGCTTTTACTGCTGCACTGAGG
	**Reverse**	CCTCAGTGCAGCAGTAAAAGCATACACCCTAGTAGGCTCGTTATTCGTAGAGG
**ΔHRE2**	**Forward**	GCTTTTACTGCTGCACTGAGGATACAAAATGCGCGCAGGCACAACGAGAC
	**Reverse**	GTCTCGTTGTGCCTGCGCGCATTTTGTATCCTCAGTGCAGCAGTAAAAGC
**ΔHRE2**	**Forward**	CGCCCCGGCGCGGGCTCGGGATACACAGAGCCGGCGAGCGAGCAGC
	**Reverse**	GCTGCTCGCTCGCCGGCTCTGTGTATCCCGAGCCCGCGCCGGGGCG

shHIF plasmids

		Sequence
**shControl**	**Forward**	GTACCTCCCTACATCCCGATCGATGATCAAGAGTCATCGATCGGGATGTAGGTTTTTGGAAA
	**Reverse**	AGCTTTTCCAAAAACCTACATCCCGATCGATGACTCTTGATCATCGATCGGGATGTAGGGAG
**shHIF1α**	**Forward**	GTACCTCCTGATGACCAGCAACTTGATCAAGAGTCAAGTTGCTGGTCATCAGTTTTTGGAAA
	**Reverse**	AGCTTTTCCAAAAACTGATGACCAGCAACTTGACTCTTGATCAAGTTGCTGGTCATCAGGAG
**shHIF2α**	**Forward**	GTACCTCCAGCATCT TTGATAGCAGTTCAAGAGACTGCTATCAAAGATGCTGTTTTTGGAAA
	**Reverse**	AGCTTT TCCAAAAACAGCATCTTTGATAGCAGTCTCTTGAACTGCTATCAAAGATGCTGGAG

HIF UTR cloning primers

		Sequence
**HIF1α**	**Forward**	GACTCTCGAGGCTTTTTCTTAATTTCATTCC
	**Reverse**	AGTCGCGGCCGCGCCTGGTCCACAGAAGATG
**HIF2α**	**Forward**	GACTCTCGAGGCCAGGCCTTCTACCTGGGC
	**Reverse**	AGTCGCGGCCGCCAGTGGTAGGATCAGAATAC

EMSA primers

		Sequence
**LIMD1 wild type HRE (LIMD1)**	**Forward**	GGGCTCGGGACGTGCAGAGCCGGC
	**Reverse**	GCCGGCTCTGCACGTCCCGAGCCC
**LIMD1 mutant HRE (mLIMD1)**	**Forward**	GGGCTCGGGAataatAGAGCCGGC
	**Reverse**	GCCGGCTCTattatTCCCGAGCCC
**PHD2 wild type HRE (LIMD1)**	**Forward**	GCCGTGGTGTACGTGCAGAGCGCG
	**Reverse**	CGCGCTCTGCACGTACACCACGGC
**PHD2 mutant HRE (mLIMD1)**	**Forward**	GCCGTGGTGTAataatAGAGCGCG
	**Reverse**	CGCGCTCTattatTACACCACGGC

ChIP primers

		Sequence
**LIMD1**	**Forward**	CAGGCCTGGGGGCAGGAG
	**Reverse**	GGGACGCGGAAGTGAGTG
**PHD2**	**Forward**	CGGGTCGCCGCGGGGCCGTGG
	**Reverse**	GGAGGAGCGCAGGGCATACGGGCG
**PHD3**	**Forward**	CGTGGAGGACTGGCTCTAAG
	**Reverse**	GGTGTGCTCGGGTGTG

Table S10 continued: Primers

qRT-PCR primers

		5'-3' Primer Sequences	
**Gene symbol**	**Gene name**	**Forward**	**Reverse**
ALDOA	Aldolase A	GCAAACGTTCTTGCACGCTA	TACTTGGTGGAGCAGCTGTG
ALDOC	Aldolase C, fructose-bisphosphate	TAACTGGCTGCGCACAGGGAGC	ATCCGCAGGGCAATGTCAGACAA
BNIP3	BCL2/adenovirus E1B 19kDa interacting protein 3	ATGTCGTCCCACCTAGTCGAG	CTCCACCCAGGAACTGTTGAG
EDN1	Endothelin 1	CAAGCAGGAAAAGAACTCAG	CTGGTTTGTCTTAGGTGTTC
mEMCN	Endomucin (mouse)	GCTATTCACATTCACTACACC	TAAAACACCTGTGCTGTTAC
ENO1	Enolase 1	AGCGGAGCGGTGTTCAAGAT	CAGCCAGAGATACGCCCAAGAT
ERO1L	Endoplasmic reticulum oxidoreductin-1-like	AGCTGATGACATTCAGTCCCCTGA	GGTCCCTTGTAACCAGTGTAGCGC
GAPDH	Glyceraldehyde 3-phosphate dehydrogenase	ACATGTGTAAGCTGCGGCC	GTTGTGCATAGTCGCTGCTTG
HIF1A	Hypoxia-inducible factor 1, alpha	CCAGTTACGTTCCTTCGATCAGT	TTTGAGGACTTGCGCTTTCA
HIF2A	Hypoxia-inducible factor 2, alpha	GTGCTCCCACGGCCTGTA	TTGTCACACCTATGGCATATCACA
HK1	Hexokinase 1	AACCAAGGCTGAGCCGAGCT	AGCCAGAGAGCCAGGCACT
KITLG	KIT ligand	AGTGATTGTGTGGTTTCTTC	TCCTATTACTGCTACTGCTG
LDHA	Lactate dehydrogenase A	AGCCGATTCCGTTACCT	CACCAGCAACATTCATTCCA
LIMD1	LIM domain-containing protein 1	TGGGGAACCTCTACCATGAC	CACAAAACACTTTGCCGTTG
PDGFB	Platelet derived growth factor B	GGGCAGGTTATTTAATATGG	AATCAGGCATCGAGACAG
PGM2	Phosphoglucomutase 2	CCTCTTTTCTGATATAACGCCAAC	CCCTTTATCGTGAGGAGAAATG
PHD2	Prolyl hydroxylase domain protein 2	CGGCTGCGAAACCATTGGGC	CCATGGCTTTCGTCCGGCCAT
SLC2A1	Solute carrier family 2 member 1	ACCTCAAATTTCATTGTGGG	GAAGATGAAGAACAGAACCAG
SLC2A14	Solute carrier family 2 member 14	CTGTGTTCTATTACTCAACAGG	GATAGTATTAACCACACCCG
SOD2	Superoxide dismutase 2	ATTTTCTGGACAAACCTCAG	TTCCTTATTGAAACCAAGCC
VEGFA	Vascular endothelial growth factor A	CACCAAGGCCAGCACATAGGAG	GCCCACAGGGATTTTCTTGTCTTGC
VEGFB	Vascular endothelial growth factor B	GAAAGTGGTGTCATGGATAG	ATGAGCTCCACAGTCAAG
VEGFC	Vascular endothelial growth factor C	CTGGCTCAGGAAGATTTTATG	TGTTTTTACAGACACACTGG
**House Keeping**			
BTUB	Beta Tubulin	ATACCTTGAGGCGAGCAAAA	CTGATCACCTCCCAGAACTTG
RNPII	RNA Polymerase II	GACACAGGACCACTCATGAAGT	GTGCGGCTGCTTCCATAAG

Table S11: Antibodies

Key	
**IB**	Immunoblotting
**IP**	Immunoprecipitation
**IHC**	Immunohistochemistry
**EMSA**	Electrophoretic mobility shift assay
**ChIP**	Chromosome immunoprecipitation

Antibody	Supplier	Cat #	Application
anti-LIMD1	Cell Signaling	#13245	**IB/IP/IHC**
anti-HIF1α	BD Biosciences	#610959	**IB/EMSA/ChIP**
anti-HIF2α	Novus Biologicals	#NB100-122	**IB/EMSA**
anti-PHD2	Abcam	#ab4561	**IB**
anti-β-actin	Sigma	#A1978	**IB**
anti-VHL	BD Biosciences	#556347	**IB**
anti-HIF1β	Cell Signaling	#3718	**IB**
anti-Flag	Cell Signaling	#2368	**IB**
anti-p53	Santa Cruz Biotechnology	#sc-126	**IB**
HRP- goat anti-mouse	Dako	P0447	**IB**
HRP- goat anti-rabbit	Dako	P0448	**IB**
anti-VEGFA	Abcam	ab27620	**IHC**
