# Supplementary material for: A HIF–LIMD1 negative feedback mechanism mitigates the pro‐tumorigenic effects of hypoxia
Source: EMBO Mol Med. 2018 Jun 21;10(8):e8304. doi: 10.15252/emmm.201708304 (PMC6079541; doi:10.15252/emmm.201708304)
Supplement: Supplementary file 2 — Expanded View Figures PDF [file EMMM-10-e8304-s002.pdf]

Expanded View Figures

Figure EV1. *LIMD1* expression is induced in hypoxia via a HIF-1 hypoxia-responsive element (HRE).

A Small airway epithelial cells (SAEC) and human dermal fibroblasts (HDF) were incubated in hypoxia for up to 48 h, total protein extracted and analysed by Western blot.

B Densitometric analysis of LIMD1 protein expression in (A), normalised to β-actin loading control and 0-h hypoxic time-point.

C *PHD2* mRNA expression is upregulated by hypoxia. A549, HEK293, HeLa and U2OS cells were incubated in hypoxia for up to 48 h, RNA extracted and *PHD2* mRNA expression analysed by qRT–PCR.

D *PHD1* mRNA expression is unchanged by hypoxia. qRT–PCR analysis of *PHD1* mRNA in the indicated cell lines, as in (C).

E–G The indicated cell lines were incubated in normoxia or 4- or 24-h hypoxia, total protein extracted and PHD2, HIF-1α or HIF-2α protein expression quantified by densitometry and normalised to β-actin loading control.

H The *LIMD1* promoter contains three putative HRE elements. The 2-kb upstream region of the *LIMD1* promoter was scrutinised *in silico* for predicted HIF binding sites using MatInspector software as previously described (Foxler *et al,* 2011).

I A 2-kbp region of the *LIMD1* promoter was cloned into a pGL4 Renilla luciferase plasmid, and a series of 10 consecutive internal deletions were constructed (Δ1–10); the positions of the three *in silico* HRE elements are indicated.

J Reporter constructs in (I) were expressed in U2OS cells and exposed to hypoxia for 24 h. Data shown are normalised to vector only (VO) control. The Δ3 mutation inhibited hypoxic induction of *LIMD1* transcription compared to the other nine analysed; significance values for all other constructs are omitted for clarity.

Data information: Unless otherwise stated, data shown are mean ± SEM, $n = 3$, *$P < 0.05$, **$P < 0.01$, n.s. = not significant, according to the Student's *t*-tests (B; comparing means to the theoretical value of 1) or Holm–Šidák *post hoc* tests, comparing time-points within each cell line, following significant main effects/interactions of a mixed-model ANOVA (C–E). See Appendix Table S6 for a summary of statistical analysis.
Source data are available online for this figure.

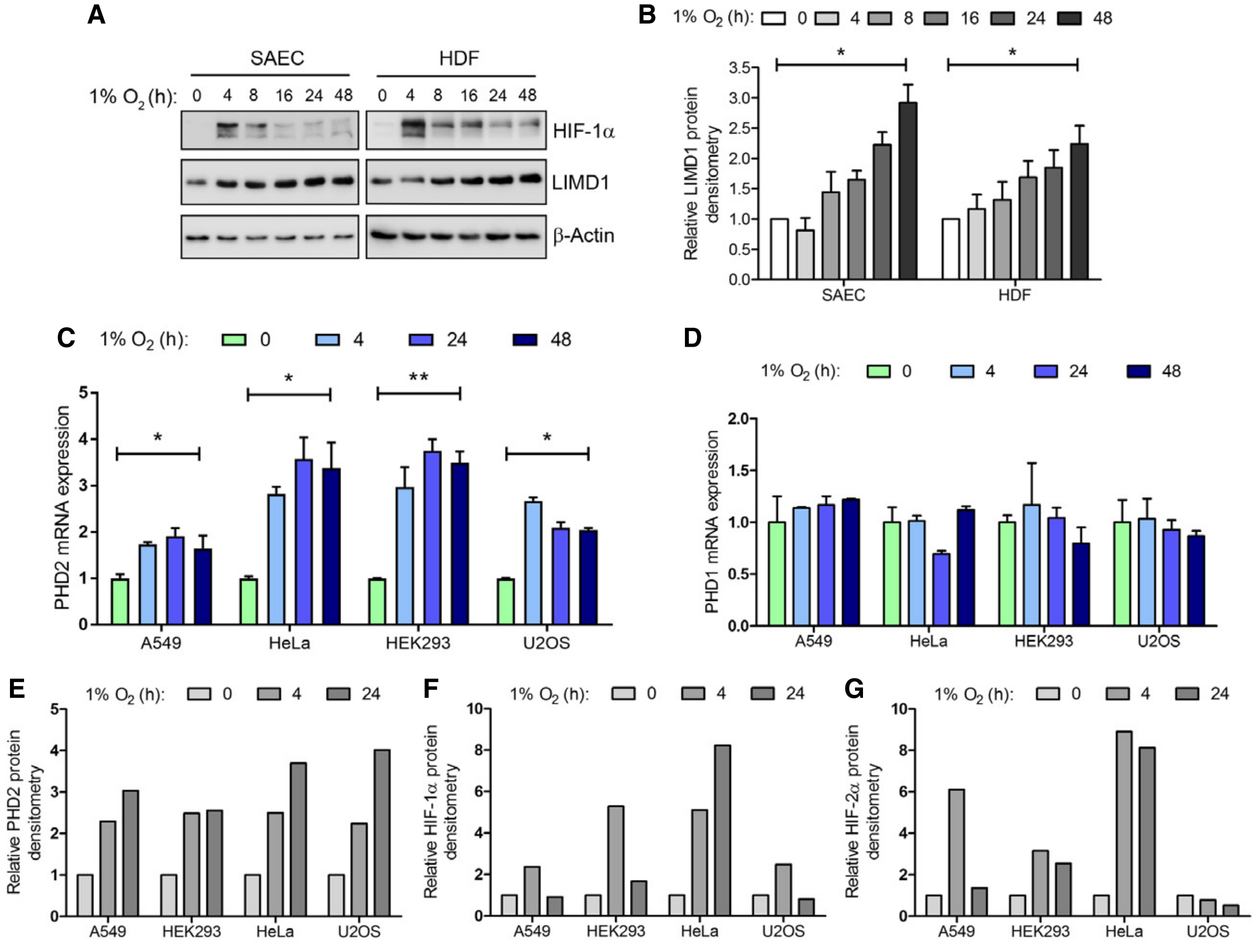

H

	Matrix Family	Detailed Family Information	Matrix	Start position	End position	Anchor position	Core sim.	Matrix sim.	Sequence
HRE 1	V$HIFF	Hypoxia inducible factor, bHLH/PAS protein family	V$ARNT.01	559	575	567	1	0.965	ctagggcaCGTGctttt
HRE 2	V$HIFF	Hypoxia inducible factor, bHLH/PAS protein family	V$HRE.01	584	600	592	1	0.915	actgaggaCGTGaaatg
HRE 3	V$HIFF	Hypoxia inducible factor, bHLH/PAS protein family	V$HRE.01	1262	1278	1270	1	0.931	gctcgggaCGTGcagag

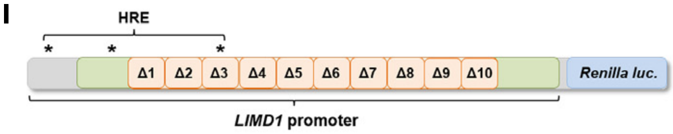

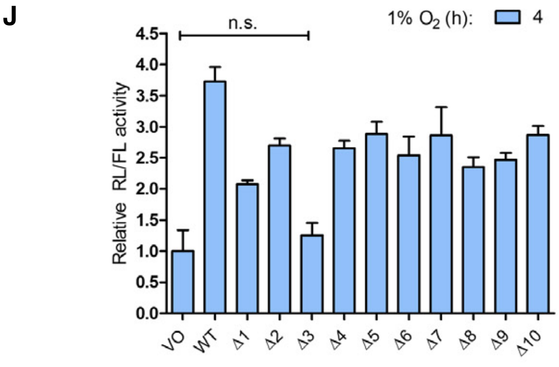

Figure EV1.

© 2018 The Authors

Figure EV2. *LIMD1* hypoxic induction occurs via HIF-1 and facilitates formation of a hypoxic HIF-1 degradation complex.

A HIF-1 but not HIF-2 is responsible for the hypoxic increase in *LIMD1* expression. HIF-1α or HIF-2α was knocked down in U2OS cells using transient shRNA-expressing plasmids. The wild-type *LIMD1* promoter-driven firefly luciferase was co-transfected with a Renilla normalisation plasmid into these cells and exposed to up to 24-h hypoxia. Resultant luciferase activity was assayed and normalised to Renilla. Data are displayed normalised to the normoxic value for each shRNA knockdown line.

B–D Hypoxic induction of LIMD1 is impaired upon knockdown of HIF-1α. Western blot analysis for the indicated proteins from HeLa, A549 and SAEC transfected with the indicated siRNA (40 nM) for 48 h prior to exposure to hypoxia for 24 h.

E siRNA-mediated depletion of HIF-1α but not HIF-2α reduces LIMD1 expression in both normoxia and hypoxia. qRT–PCR analysis of *LIMD1* mRNA from HeLa cells transfected with the indicated siRNA (40 nM) and maintained in normoxia (20% O_2) or exposed to hypoxia (1% O_2) for 24 h.

F–I Knockdown of HIF-1α but not HIF-2α significantly reduces *LIMD1* mRNA expression in both normoxia and hypoxia. qRT–PCR analysis of the indicated mRNA from cellular extracts in (B) and Fig 2C.

J LIMD1 endogenously complexes with PHD2, VHL, HIF-1α and HIF-2α. Western blot analysis of endogenous LIMD1 immunoprecipitated from A549 cells in either normoxia or following 24-h hypoxia.

Data information: Unless otherwise stated, data shown are mean ± SEM, $n = 3$, $^*P < 0.05$, $^{**}P < 0.01$, according to Holm–Šidák *post hoc* tests, comparing siRNA treatment within each time-point, following significant main effects/interactions of a mixed-model ANOVA (A) or Holm–Šidák-corrected one-sample Student's *t*-tests (E to I; comparing means to the theoretical value of 1). See Appendix Table S7 for a summary of statistical analysis.
Source data are available online for this figure.

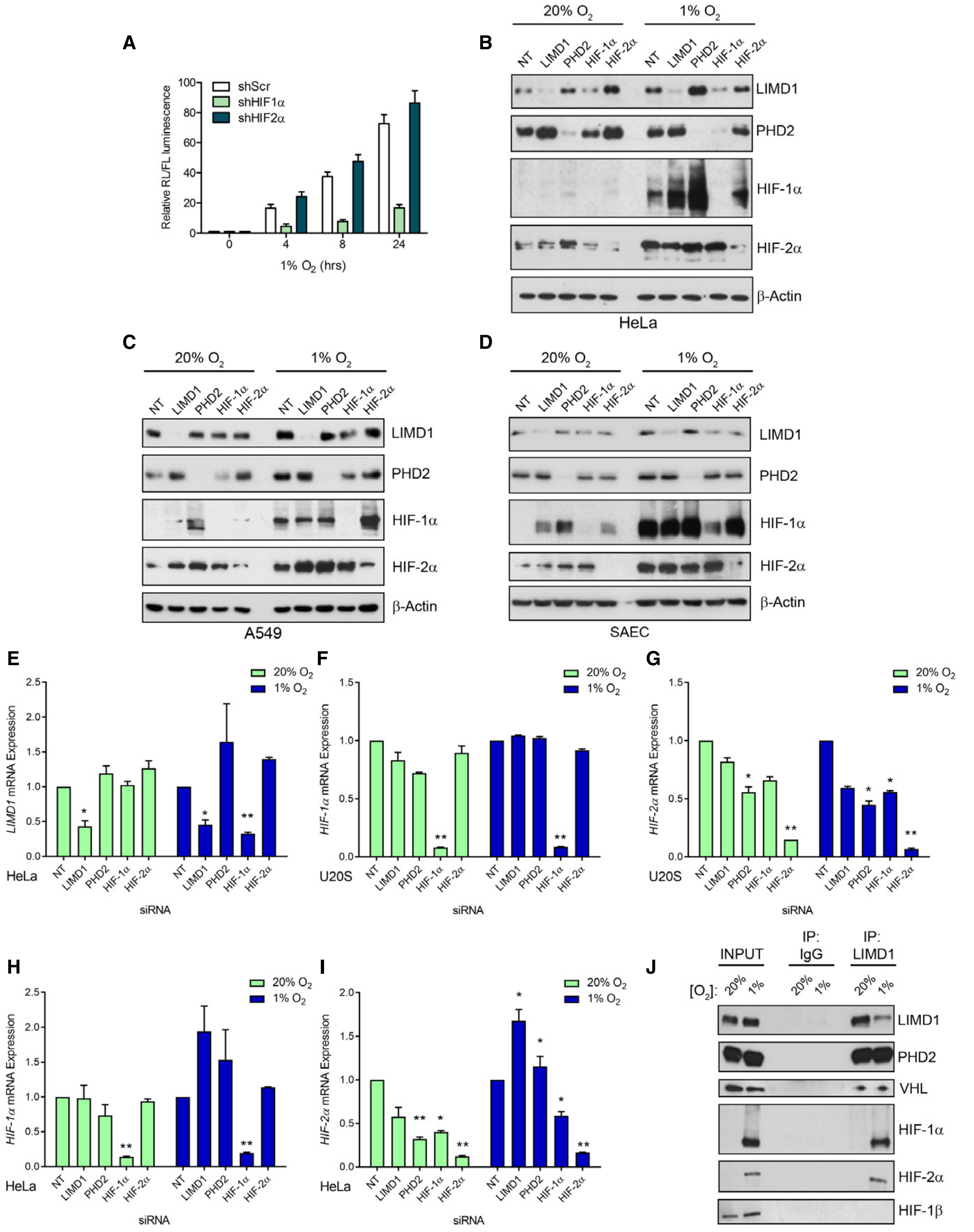

Figure EV2.

 © 2018 The Authors

Figure EV3. Hypoxic induction of LIMD1 represses HIF-1 transcriptional activity.

A Sequence verification of mutation of the HRE motif within the *LIMD1* promoter in the HRE^{mut} lentiviral cassette.

B, C Densitometric quantification of LIMD1 and Flag (LIMD1) protein expression in U2OS HRE isogenic cell lines (Fig 3B, mean ± SEM, n = 3). Expression of each protein was normalised to β-actin loading control and the 0-h time-point.

D, E Densitometric quantification of LIMD1 protein expression in HeLa (D) and SAEC (E) HRE isogenic cell lines (Fig 3B, mean ± SEM, n = 3). Expression of each protein was normalised to β-actin loading control and the 0-h time-point.

F *HIF1A* mRNA increases after 24 h hypoxia in the HRE^{mut} line. qRT–PCR analysis of RNA from HRE^{wt} and HRE^{mut} cells exposed to hypoxia for the indicated times. *HIF1A* mRNA expression was normalised to the RPII housekeeping gene.

G Hypoxic *LIMD1* induction decreases HRE-luciferase activity in hypoxia. The isogenic cell lines were co-transfected with a synthetic HRE-luciferase (pNL-HRE) and pGL3 Firefly normalisation plasmid, prior to exposure to hypoxia. Luciferase activity was assayed and normalised against HRE activity in the HRE^{wt} line. After 24-h hypoxic exposure, the HRE^{mut} line had significantly increased luciferase activity compared to the HRE^{wt} line. n = 3, $^{**}P < 0.01$.

H–K Hypoxic *LIMD1* induction decreases endogenous HIF-driven gene expression in hypoxia. qRT–PCR analysis of RNA extracted from the U2OS (I), HeLa (J-K) and SAEC (L) isogenic cell lines following normoxic or hypoxic (24 h) exposure. The HRE^{mut} line had significantly increased HIF-1-driven gene expressions compared to the HRE^{wt} line.

L Hypoxic *LIMD1* induction decreases VEGF-A secretion in hypoxia. The isogenic HeLa cell lines were incubated in hypoxia for 48 h, and VEGF-A secretion was quantified by ELISA.

Data information: Unless otherwise stated, data shown are mean ± SEM, n = 3, $^{*}P < 0.05$, $^{***}P < 0.001$, according to Holm–Šidák *post hoc* tests, comparing genotype within each time-point, following significant main effects/interactions of a mixed-model ANOVA (B–E) or standard two-way ANOVA (F, G & J), Holm–Šidák-corrected one-sample Student's *t*-tests (H and I; comparing means to the theoretical value of 1), or separate Mann–Whitney *U*-tests, comparing WT and MT within each gene (K). See Appendix Table S8 for a summary of statistical analysis.

© 2018 The Authors

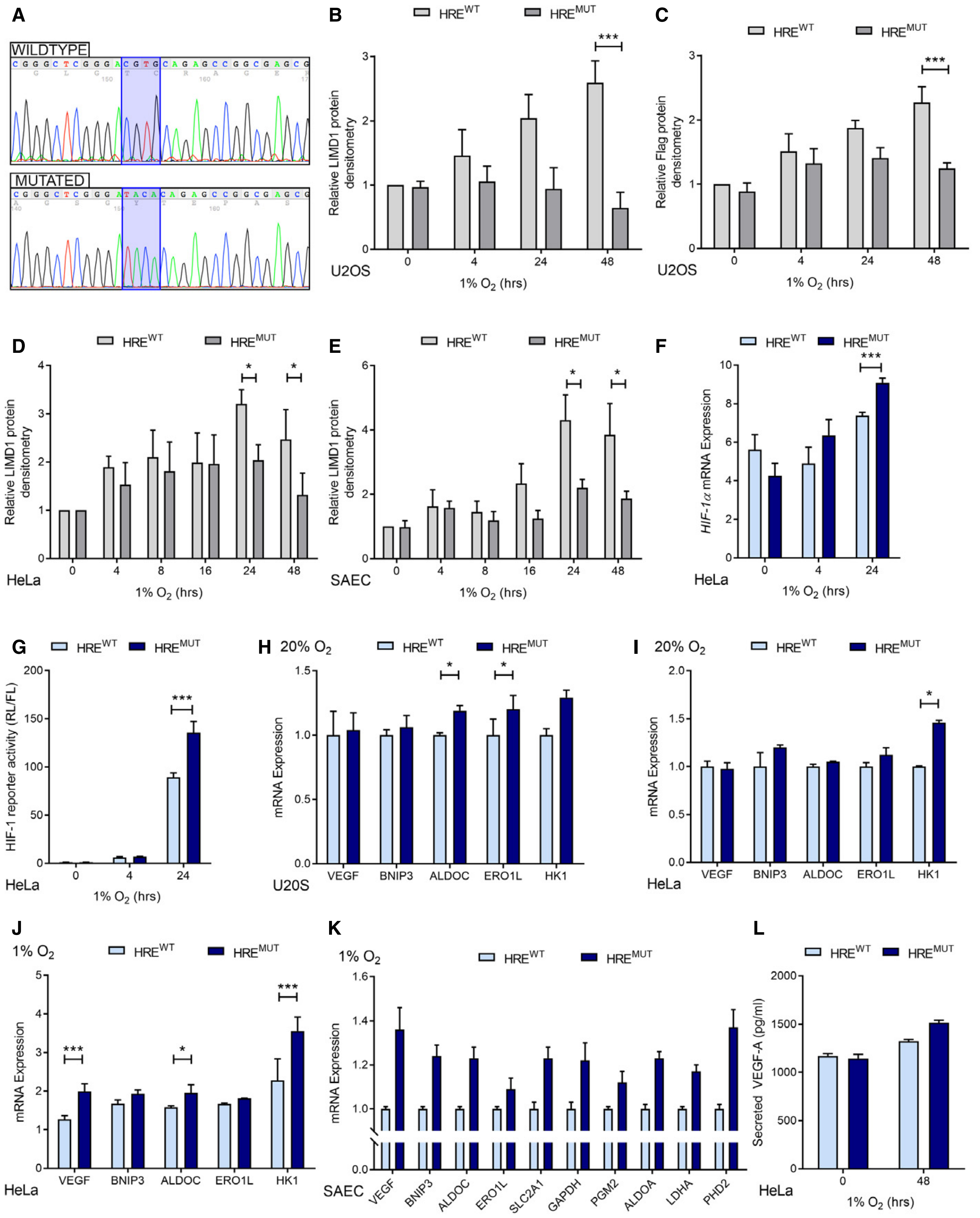

Figure EV3.

 © 2018 The Authors

Figure EV4. LIMD1 hypoxic induction represses HIF-1 target gene expression *in vivo*.

A Increased hypoxic LIMD1 expression does not affect 2D growth rates. A549 isogenic cell lines were seeded into 6-well plates and counted every 2 days to determine their proliferation rate in both normoxia and hypoxia.

B Increased hypoxic LIMD1 expression does not affect colony formation ability. A549 isogenic cell lines were seeded into 6-well plates at 100 cells/well and colony formation allowed to proceed for 10 days in either normoxia or hypoxia. Cells were then fixed and stained with crystal violet for counting.

C–O qRT–PCR analysis of the indicated HIF-responsive genes from HRE^{wt}- and HRE^{mut}-derived xenografts. Eight- to 12-week-old female SCID/beige mice were injected subcutaneously with 5×10^9 HRE^{wt} or HRE^{mut} A549 cells and subsequent xenograft growth measured until the first measurement exceeded the legal limit of 1.44 cm^2. Xenografts were removed and snap frozen in TriPure. Xenografts were homogenised and followed by aqueous phase RNA extraction. RNA expression was analysed by qRT–PCR and normalised to the RPII housekeeping gene. $n = 15$ (HRE^{wt}) and $n = 14$ (HRE^{mut}).

Data information: Unless otherwise stated, data shown are mean ± SEM, $n = 3$, *$P < 0.05$, **$P < 0.01$, ***$P < 0.001$, n.s. = not significant, according to separate Welch-corrected Student's t-tests for each gene. See Appendix Table S9 for a summary of statistical analysis.

© 2018 The Authors

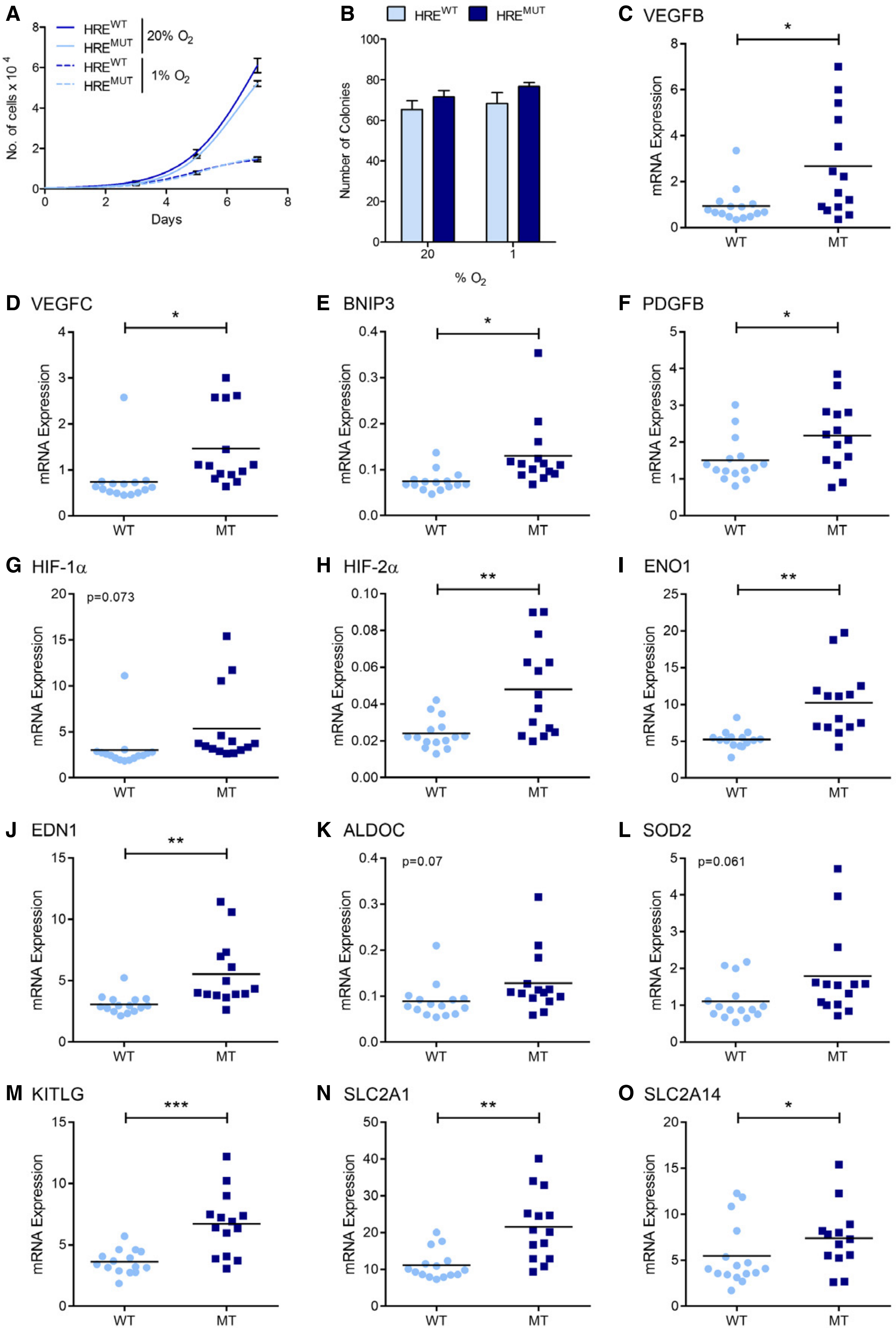

Figure EV4.

 © 2018 The Authors

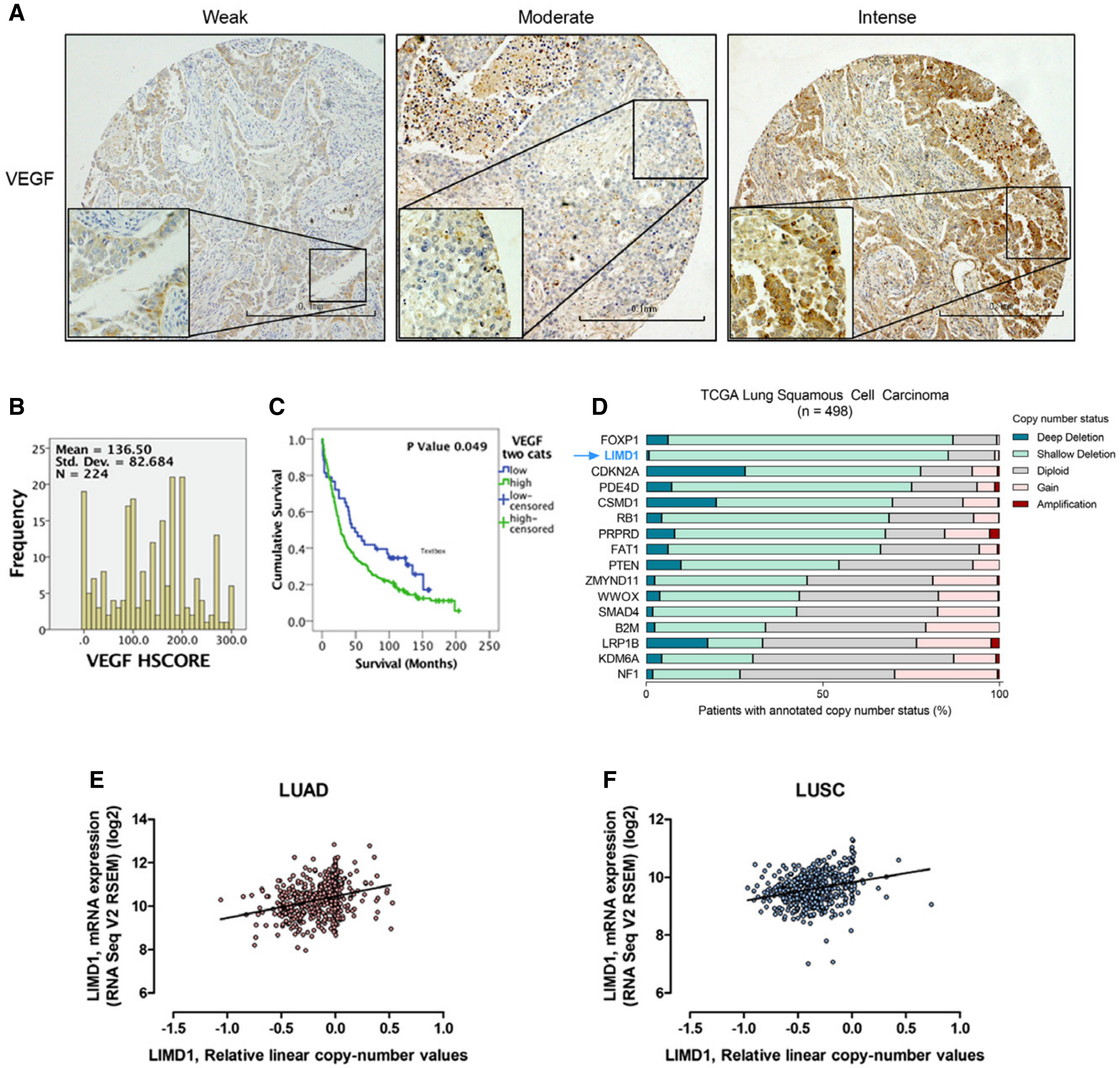

Number of XY Pairs	512
Pearson r	0.2877
95% confidence interval	0.2062 to 0.3653
P value (two-tailed)	< 0.0001
P value summary	****
Is the correlation significant? (alpha=0.05)	Yes
R square	0.08278

Number of XY Pairs	498
Pearson r	0.2698
95% confidence interval	0.1863 to 0.3494
P value (two-tailed)	< 0.0001
P value summary	****
Is the correlation significant? (alpha=0.05)	Yes
R square	0.07279

Figure EV5. HIF target gene VEGF-A expression is a poor prognostic indicator in lung adenocarcinoma and squamous cell carcinoma, where *LIMD1* is frequently lost.

A, B (A) Representative immunohistochemistry (IHC) staining of cores for VEGF-A that were scored in the 276 patient sample TMA to ascertain relative expression (H-score) within the cohort (B). Scale bar 100 μm; 20 μm on zoom panel.

C Kaplan–Meier analysis identifies patients stratified as having high VEGF-A expression (intense staining) exhibit poorer overall survival compared to low (weak staining).

D Copy number alterations of *LIMD1* and other validated tumour suppressor genes from a lung squamous cell carcinoma (LUSC) cohort (n = 498), publically available from TCGA.

E, F (E) Regression analysis performed upon a lung adenocarcinoma (LUAD) and (F) squamous cell carcinoma (LUSC) cohort from TCGA demonstrates significant correlation between *LIMD1* copy number and mRNA expression.

© 2018 The Authors
