# Supplementary figures and images for: A HIF–LIMD1 negative feedback mechanism mitigates the pro‐tumorigenic effects of hypoxia
Source: EMBO Mol Med. 2018 Jun 21;10(8):e8304. doi: 10.15252/emmm.201708304 (PMC6079541; doi:10.15252/emmm.201708304)

Source data: Figure EV1

Figure EV1A

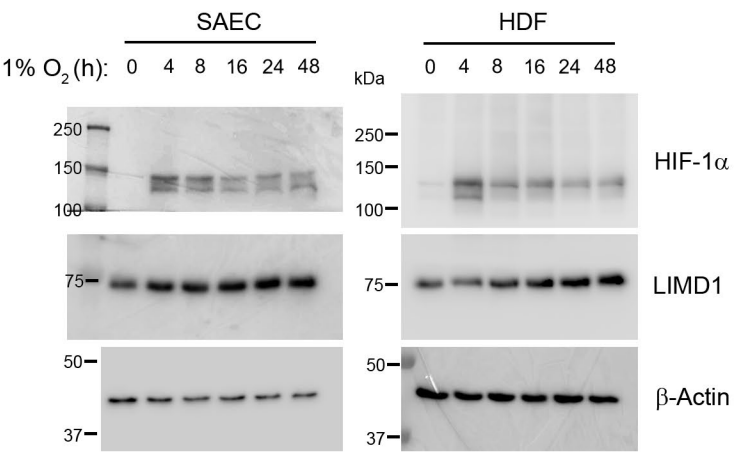

Supplement: Supplementary file 4 — Source Data for Expanded View [file EMMM-10-e8304-s009.zip › EMM-2017-08304-source_data/Source_data_EV1.pdf]

Source data: Figure EV2

Figure EV2B

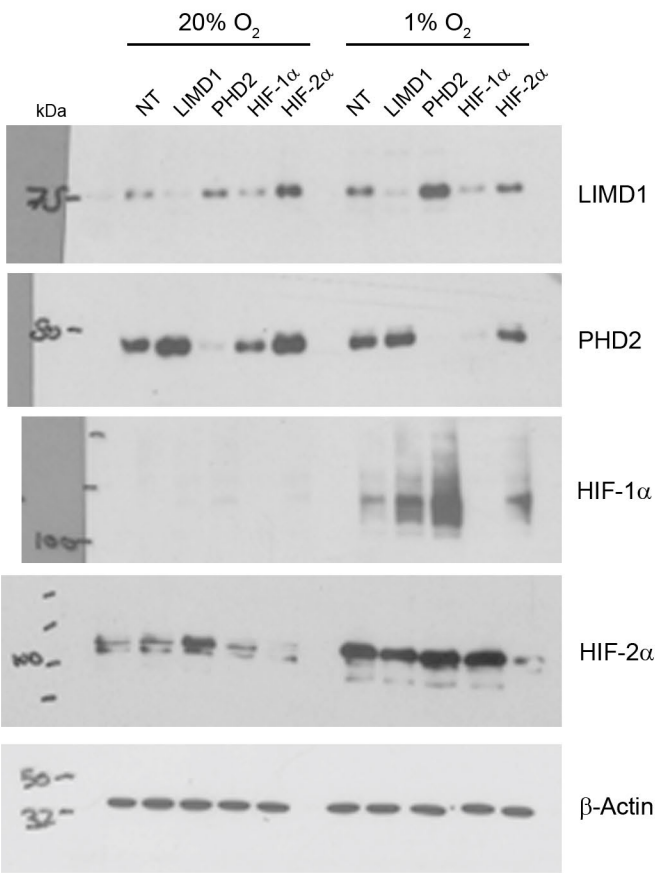

Figure EV2C

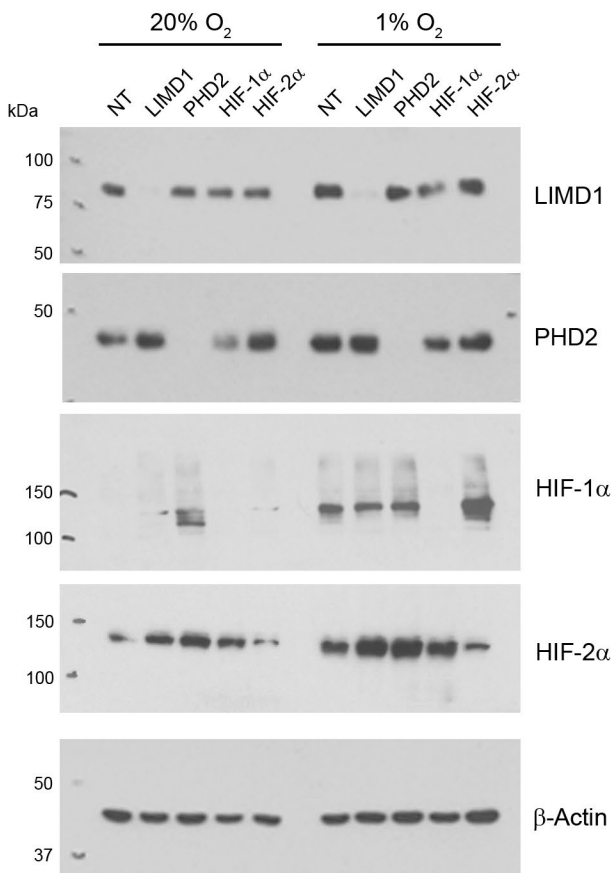

Figure EV2D

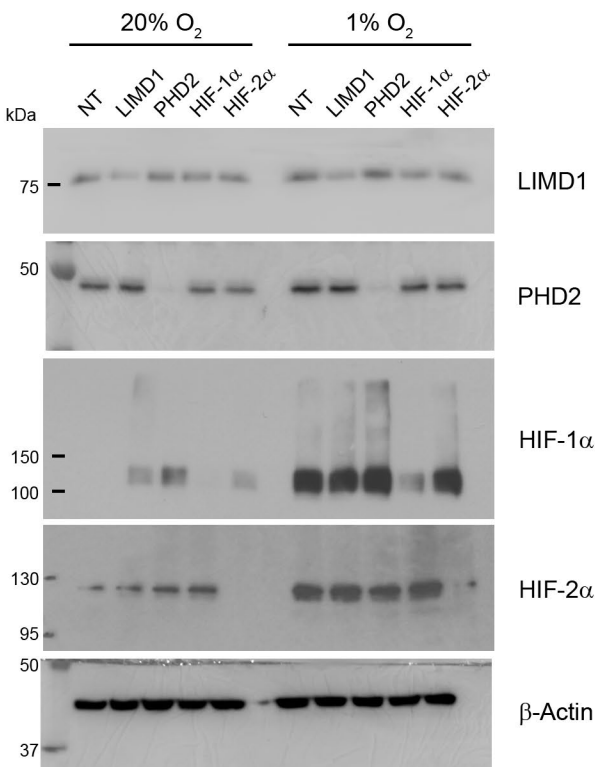

Figure EV2J

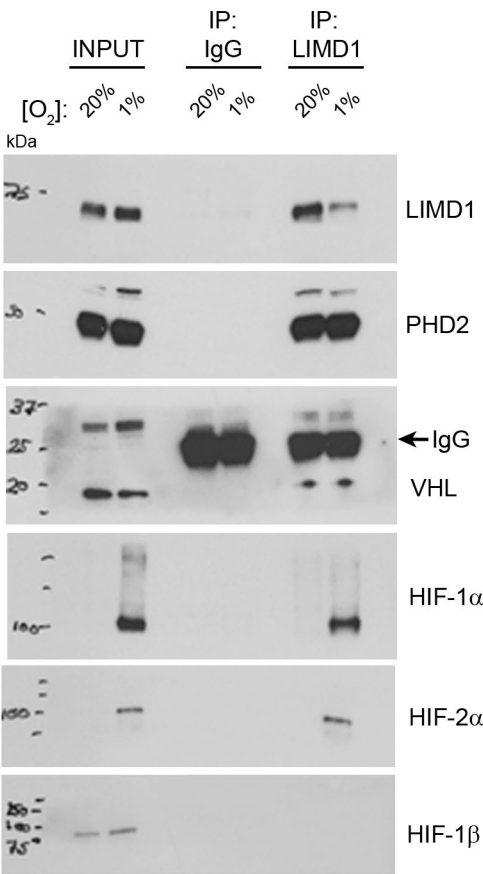

Supplement: Supplementary file 4 — Source Data for Expanded View [file EMMM-10-e8304-s009.zip › EMM-2017-08304-source_data/Source_data_EV2.pdf]

Source data: Figure 1

Figure 1B

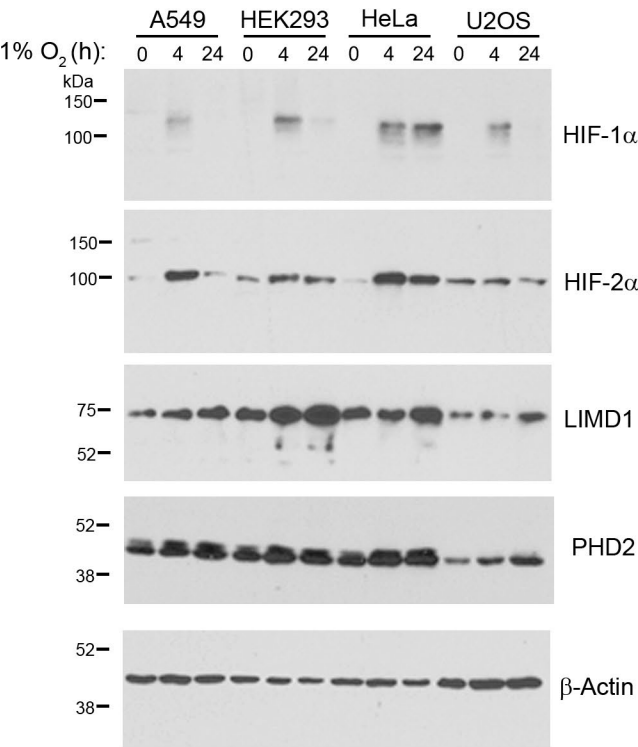

Supplement: Supplementary file 6 — Source Data for Figure 1 [file EMMM-10-e8304-s004.pdf]

Source data: Figure 2

Figure 2B

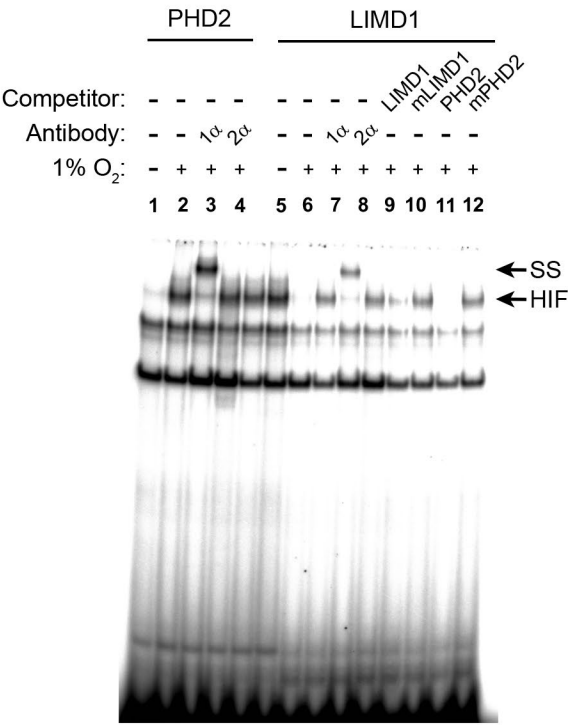

Figure 2D

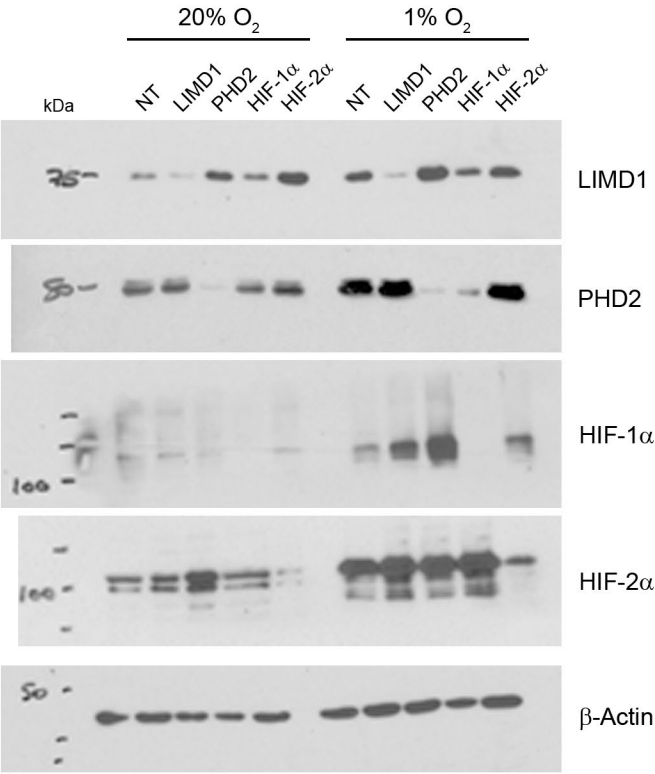

Figure 2E

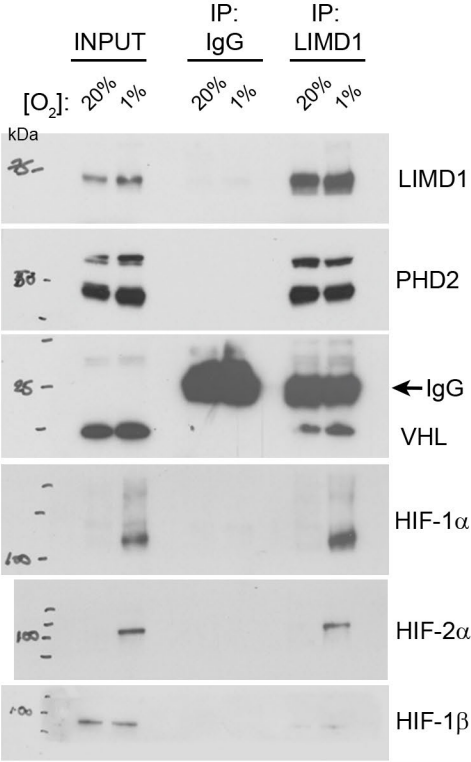

Supplement: Supplementary file 7 — Source Data for Figure 2 [file EMMM-10-e8304-s005.pdf]

Source data: Figure 3

Figure 3B

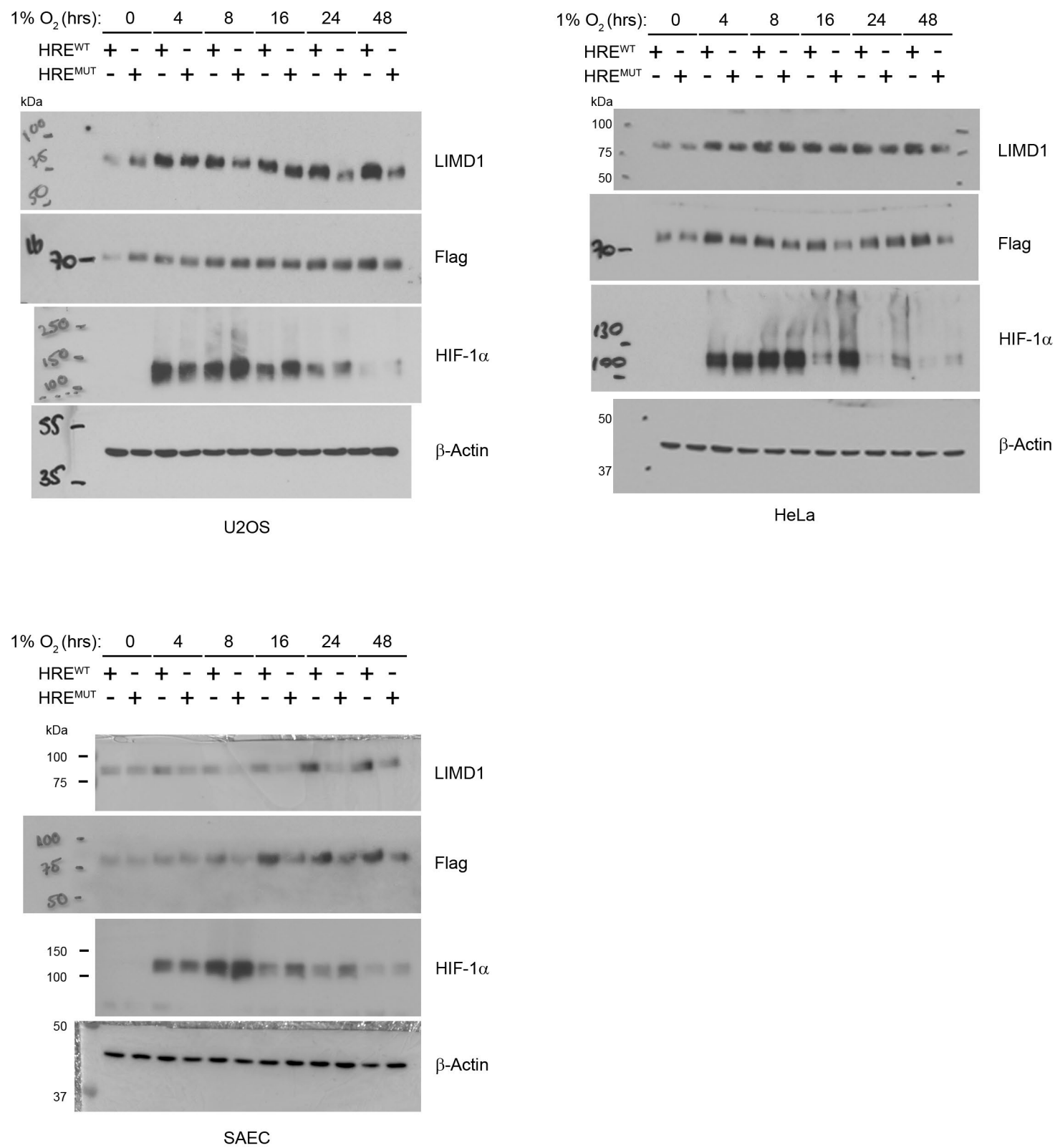

Supplement: Supplementary file 8 — Source Data for Figure 3 [file EMMM-10-e8304-s006.pdf]

Source data: Figure 4

Figure 4A

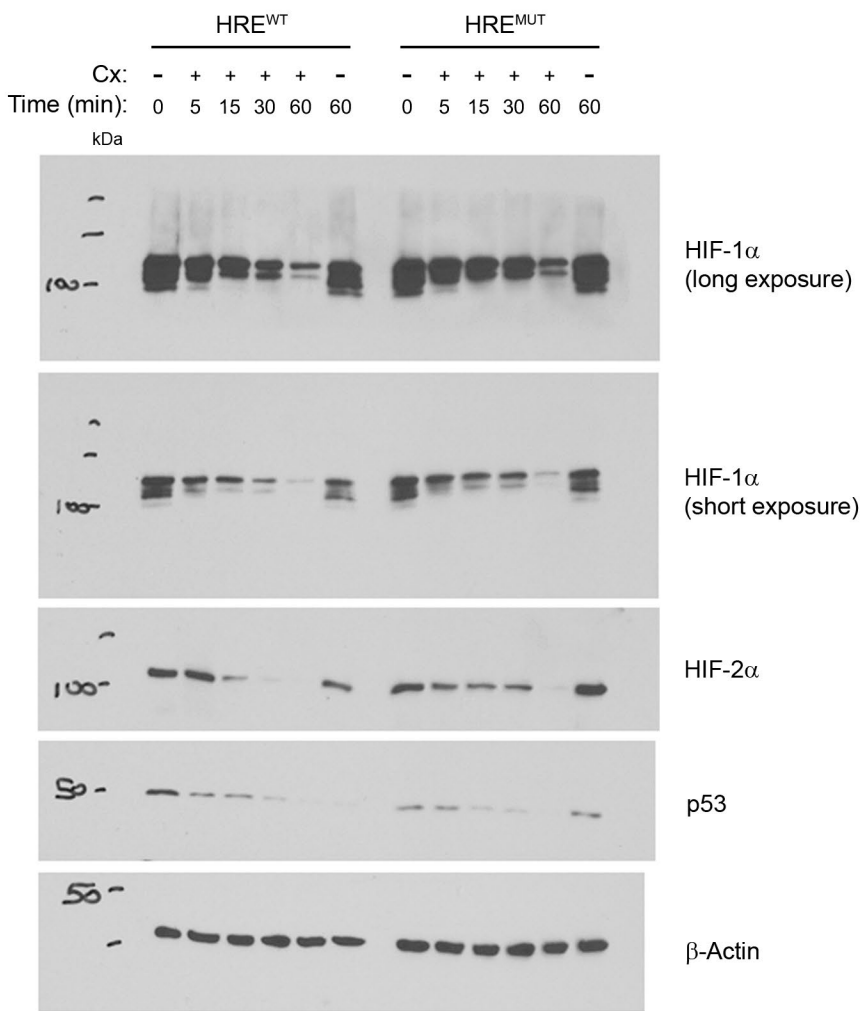

Figure 4C

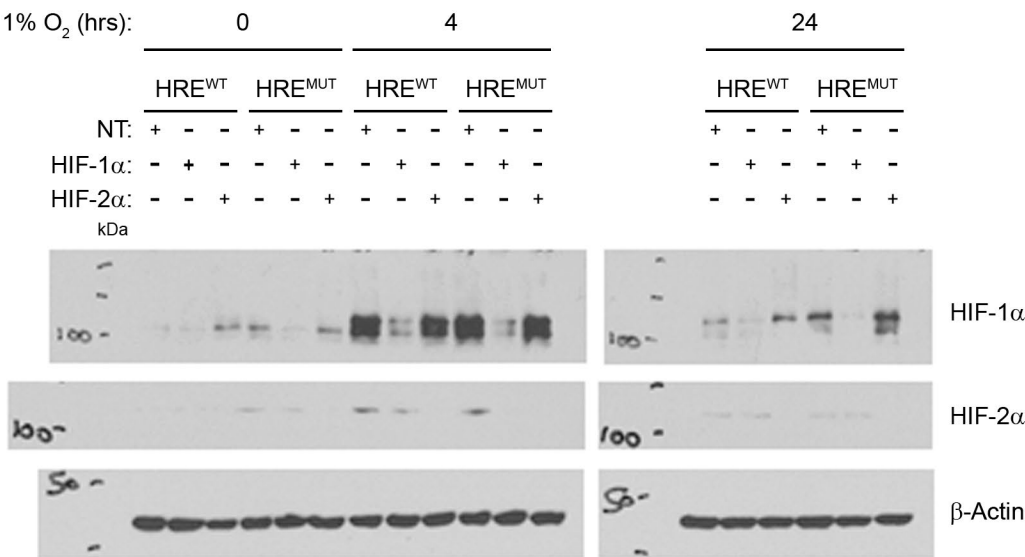

Supplement: Supplementary file 9 — Source Data for Figure 4 [file EMMM-10-e8304-s007.pdf]

Source data: Figure 5

Figure 5E

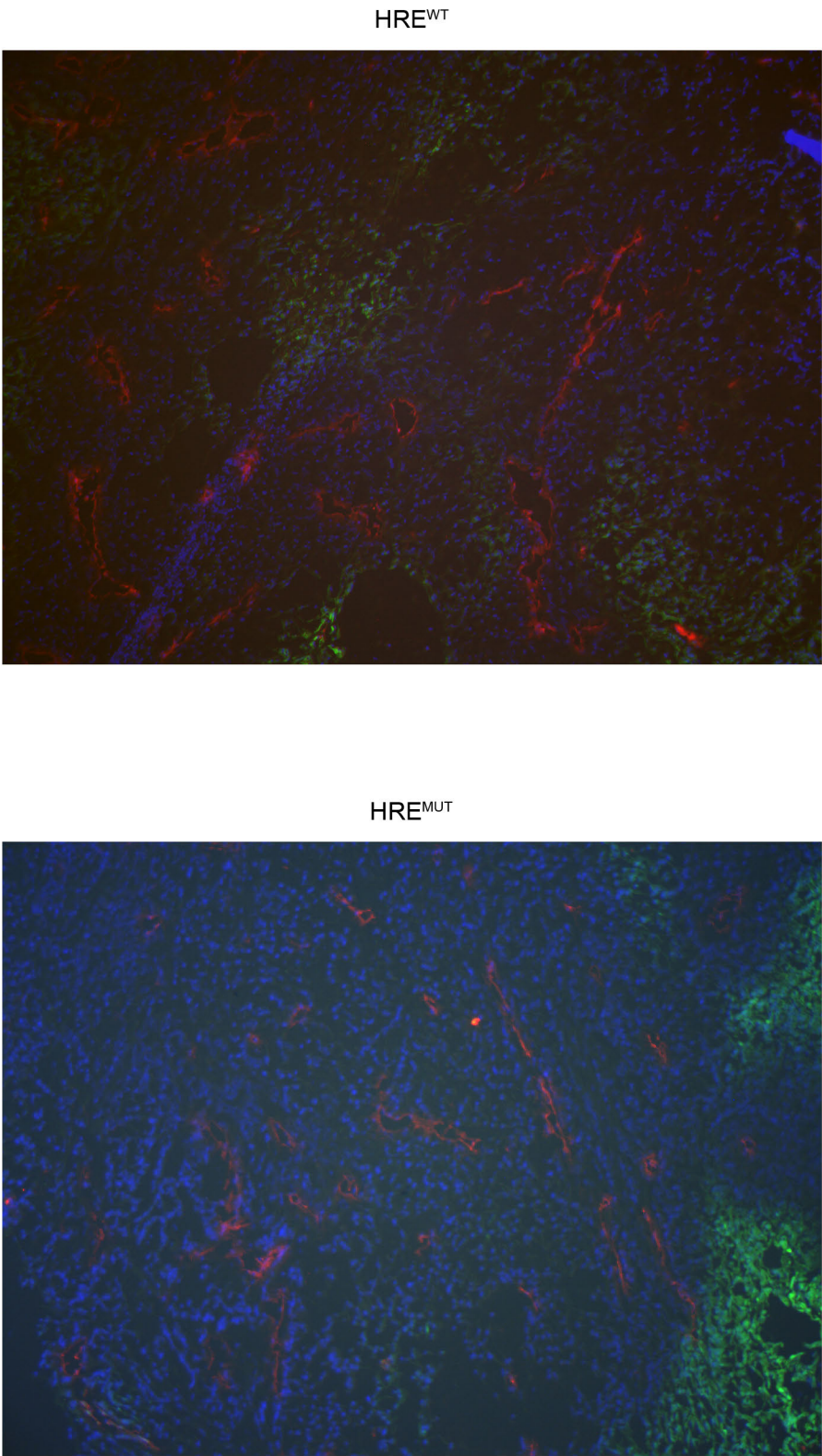

Supplement: Supplementary file 10 — Source Data for Figure 5 [file EMMM-10-e8304-s008.pdf]
